# Bi-Assisted Double-Sided ZnO Nanorod Architectures for Fast and Geometry-Enhanced X‑Ray Scintillation

**DOI:** 10.1021/acsaom.6c00409

**Published:** 2026-08-25

**Authors:** Sinem V. Kurudirek, Anna Erickson, Paul J. Sellin, Benjamin J. Lawrie, Tanya A. Balandin, Luis Stand, Charles L. Melcher, Jason D. Azoulay, Nolan E. Hertel, Murat Kurudirek

**Affiliations:** † Nuclear and Radiological Engineering Program, G. W. Woodruff School of Mechanical Engineering, 1372 Georgia Institute of Technology, Atlanta, Georgia 30332, United States; ‡ School of Mathematics and Physics, 3660 University of Surrey, Guildford GU2 7XH, U.K.; § Center for Nanophase Materials Sciences, 6146 Oak Ridge National Laboratory, Oak Ridge, Tennessee 37831, United States; ∥ Materials Science and Technology Division, Oak Ridge National Laboratory, Oak Ridge, Tennessee 37831, United States; ⊥ School of Chemistry and Biochemistry, School of Materials Science and Engineering, Center for Organic Photonics and Electronics, Georgia Institute of Technology, Atlanta, Georgia 30332, United States; # Scintillation Materials Research Center, 4285 University of Tennessee, Knoxville, Tennessee 37996, United States; ¶ Department of Nuclear Engineering, University of Tennessee, Knoxville, Tennessee 37996, United States; ∇ Department of Materials Science and Engineering, University of Tennessee, Knoxville, Tennessee 37996, United States; ○ Department of Electricity and Energy, Technical Sciences Vocational College, 37503 Ataturk University, 25240 Erzurum, Turkey

**Keywords:** ZnO:Bi nanorods, double-sided architecture, X-ray scintillators, radioluminescence, defect-mediated emission, transparent scintillators

## Abstract

Bi-assisted ZnO nanorod (NR) arrays are investigated as thin, transparent, and fast nanostructured scintillator architectures for X-ray detection. Vertically aligned ZnO:Bi NR arrays were grown hydrothermally on fused-silica substrates in single-sided, refresh-grown, and double-sided configurations to investigate the effects of Bi-assisted growth, effective scintillating volume, and collection geometry on the measured luminescence response. Structural and morphological analyses show that moderate Bi-assisted growth preserves the preferential *c*-axis-oriented wurtzite ZnO structure while producing dense, high-aspect-ratio NR arrays. Optical spectroscopy reveals a visible-emission-dominant response, with broad yellow–orange photoluminescence centered at ∼580–600 nm and weak near-band-edge emission. Cathodoluminescence imaging and spectral mapping further show that the visible emission is spatially enhanced at NR edge/sidewall regions, supporting a surface/defect-mediated recombination mechanism. X-ray photoelectron spectroscopy and energy-dispersive X-ray spectroscopy analyses of representative ZnO:Bi samples support the presence of Bi species and surface/defect-related oxygen contributions. Time-resolved PL measurements yield a fast amplitude-weighted average lifetime of ∼5.3 ns, indicating rapid defect-mediated visible recombination. Under 45 kV X-ray tube voltage, double-sided ZnO:Bi NR arrays exhibit broad radioluminescence centered at ∼600 nm in both reflection and transmission geometries. Under the investigated measurement conditions, the integrated detected RL intensity in transmission geometry is approximately 6–8 times that measured in reflection geometry. Among the double-sided samples, ZnO:Bi (5%) provides the highest measured transmission-mode RL intensity, whereas higher nominal Bi loading reduces the response, consistent with increased disorder and nonradiative loss pathways. The spectral separation between strong near-band-edge absorption and yellow–orange RL emission may reduce reabsorption losses and facilitate visible-light transport through the NR architecture. These results demonstrate that Bi-assisted double-sided ZnO NR architectures provide a promising platform for fast, transparent, and geometry-enhanced X-ray scintillators.

## Graphical abstract

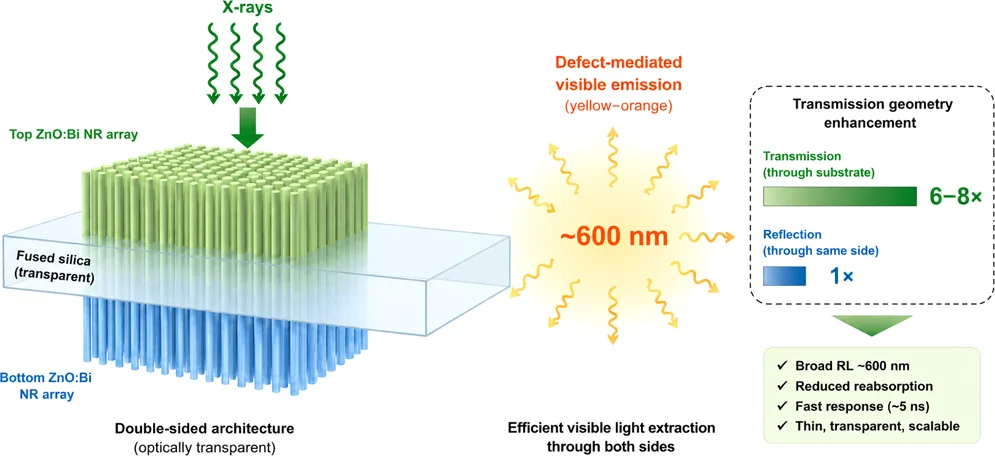

## 1 Introduction

Radiation detection plays a critical role in a broad range of applications, including medical imaging, synchrotron science, high-energy physics, homeland security, and nuclear monitoring.^1^ In indirect radiation detection systems, scintillators convert high-energy ionizing radiation into ultraviolet or visible photons that are subsequently detected by photomultiplier tubes or solid-state photodetectors.^2^ For advanced imaging and timing technologies, scintillator materials with fast response, high spatial resolution, low afterglow, and good radiation hardness are highly desirable. In particular, ultrafast scintillators operating in the nanosecond or subnanosecond regime are of considerable interest for time-resolved X-ray imaging, synchrotron diagnostics, and high-count-rate radiation measurements.^3,4^

Among the various scintillator materials investigated to date, ZnO has attracted substantial attention owing to its wide direct band gap (∼3.37 eV), large exciton binding energy (∼60 meV), fast excitonic recombination, high radiation hardness, and compatibility with low-temperature and cost-effective synthesis routes.^5^ In addition, ZnO can be synthesized in a variety of nanoarchitectures, including nanorods (NRs), nanowires, and nanoarrays, making it particularly attractive for scintillator screens and imaging applications.^5–7^ The wide band gap, high surface-to-volume ratio, and tunable electronic and surface-defect properties of such ZnO nanoarchitectures have also enabled their extensive use in UV photodetectors, where the photoresponse can be modified through elemental doping and nanostructure engineering.^8–11^ This combination of fast recombination, radiation tolerance, low-temperature processability, and architectural tunability makes ZnO a particularly suitable platform for thin, substrate-supported scintillator screens in which the NR morphology, interaction depth, defect-mediated emission, and optical collection geometry can be engineered. However, despite these advantages, ZnO-based scintillators still face important limitations.^4^ The relatively low effective atomic number and density of ZnO reduce the interaction probability with high-energy X-rays and γ-rays, thereby limiting the deposited energy and scintillation efficiency, especially for thin active layers.^12,13^ In addition, self-absorption and defect-related carrier trapping may strongly influence the optical output and scintillation dynamics of ZnO scintillators.^1,14,15^

Vertically aligned ZnO NR architectures provide a promising strategy to address some of these limitations. Compared with planar scintillator geometries, aligned NR arrays can improve light extraction and optical guiding while reducing lateral photon scattering, which is particularly advantageous for high-spatial-resolution imaging systems.^16–19^ Increasing the NR length can also enhance the interaction volume and improve X-ray energy deposition within the active scintillation region. Previously, we demonstrated ultralong visible-emitting ZnO NR arrays with enhanced X-ray-excited luminescence and nanosecond-scale decay behavior.^4^ Nevertheless, further optimization of the NR morphology, interaction depth, and scintillation response remains necessary for improving the practical performance of ZnO-based nanoscintillators.

Defect engineering represents another critical aspect of ZnO scintillator optimization. The luminescence properties of ZnO are highly sensitive to intrinsic and surface-related defects, including oxygen-vacancy-related centers, zinc interstitials, donor-like states, and surface defect complexes.^20–24^ In ZnO nanostructures, these defects strongly affect the balance between near-band-edge and deep-level emission, as well as carrier recombination dynamics.^25–28^ Surface-sensitive cathodoluminescence (CL) studies have further shown that defect-related visible emission is often localized near NR edges and surface regions, highlighting the strong coupling between morphology, surface chemistry, and optical response.^29–33^ Accordingly, controlled defect engineering rather than complete defect suppression can provide an effective route for tailoring visible scintillation behavior and optical transport in ZnO nanoarchitectures.^4,34–37^

In this context, Bi-assisted ZnO nanostructures are particularly interesting because Bi incorporation or Bi-related growth modification may influence the crystal growth kinetics, defect landscape, optical emission characteristics, and radiation interaction behavior.^38–40^ Previous studies on Bi-containing ZnO systems have mainly focused on electrical, magnetic, photocatalytic, or conventional photoluminescence (PL) properties in nanoparticles, thin films, or irregular nanostructures.^38,41,42^ However, to the best of our knowledge, Bi-assisted vertically aligned ZnO NR arrays have not previously been investigated for X-ray scintillation or radioluminescence (RL) applications. Moreover, the integration of such arrays into double-sided, transparent-substrate-supported architectures, together with the direct comparison of reflection and transmission RL collection geometries using the same samples, has not been systematically examined.

In the present work, we report Bi-assisted vertically aligned ZnO NR scintillator architectures grown hydrothermally on fused silica substrates in both single-sided and double-sided configurations. Structural, morphological, optical, and defect-related properties were systematically investigated using X-ray diffraction (XRD), scanning electron microscopy (SEM), UV–Vis spectroscopy, PL, CL, X-ray photoelectron spectroscopy (XPS), and EPR-supported defect interpretations. The scintillation behavior was evaluated through RL measurements in both reflection and transmission geometries. Building on our previous ultralong ZnO NR scintillator structures, the present study investigates Bi-assisted vertically aligned ZnO NR arrays for X-ray scintillation by developing and comparing single-sided, refresh-grown, and double-sided architectures and by directly evaluating the geometry-dependent detected RL response of the double-sided arrays in reflection and transmission configurations. The resulting architectures exhibit broad visible RL emission and fast visible-emission dynamics with an amplitude-weighted average lifetime of ∼5 ns. Collectively, the results establish Bi-assisted growth and double-sided, substrate-supported NR integration as a distinctive strategy for developing fast and geometry-responsive ZnO-based nanoscintillators.

## 2 Experimental Section

### 2.1 Materials and Growth of Bi-Doped ZnO NR Arrays

Vertically aligned ZnO NR arrays were synthesized on fused silica substrates using a low-temperature hydrothermal method adapted from our previous reports.^4,43^ Prior to NR growth, a ZnO seed layer with a thickness of approximately 100–200 nm was deposited onto the substrates by RF magnetron sputtering under Ar plasma bombardment to promote preferential *c*-axis-oriented growth. For the hydrothermal synthesis, an aqueous precursor solution was prepared using zinc nitrate hexahydrate (Zn­(NO_3_)_2_·6H_2_O, Sigma-Aldrich) and hexamethylenetetramine (HMTA, C_6_H_12_N_4_, Sigma-Aldrich) with a molar ratio of 2:1 in deionized water. Subsequently, 0.8 M ammonium hydroxide (NH_4_OH, Sigma-Aldrich) and 5 mM polyethylenimine (PEI, (C_2_H_5_N)_n_, Sigma-Aldrich) were added to further control the ZnO NR growth process. Bismuth-assisted growth was implemented by adding bismuth­(III) nitrate pentahydrate (Bi­(NO_3_)_3_·5H_2_O) into the precursor solution at different nominal concentrations relative to the Zn precursor concentration (1%–20%).

The seeded substrates were immersed in the precursor solution and maintained at 95 °C for 25 h. During growth, zinc nitrate and HMTA supplied Zn^2+^ and OH^–^ species, respectively, while HMTA additionally acted as a buffering agent that gradually released hydroxyl ions into the solution. The addition of ammonium hydroxide reduced rapid homogeneous precipitation by coordinating free Zn ions in the solution. PEI acted as a growth-directing additive by preferentially adsorbing onto the lateral nonpolar surfaces of ZnO, thereby suppressing lateral growth and promoting preferential elongation along the *c*-axis direction.

Three different ZnO NR architectures were prepared in this study: single-side growth, refresh growth, and double-sided growth structures. In the single-side configuration, ZnO NRs were grown on one side of the substrate by using a single hydrothermal cycle. For the refresh-growth structures, the initially grown ZnO NR arrays were subjected to an additional hydrothermal growth step by using a freshly prepared precursor solution. In the double-sided architecture, ZnO NRs were sequentially grown on both sides of the fused silica substrate by using separate hydrothermal growth steps, resulting in an increased effective scintillating thickness. After synthesis, all samples were thoroughly rinsed with deionized water to remove residual precursor species and dried under ambient conditions.

### 2.2 Structural, Morphological, and Optical Characterization

The surface morphology and cross-sectional structure of the ZnO NR arrays were investigated using a field-emission scanning electron microscope (HITACHI SU8230) equipped with a cold field-emission gun for high-resolution low-voltage imaging. Crystal structure, phase identification, and preferential orientation analyses were carried out using an X-ray diffractometer (PANalytical X’Pert PRO) with Cu K_α_ radiation (λ = 1.54187 Å). The diffraction peaks were indexed according to the hexagonal wurtzite ZnO crystal structure (JCPDS, now ICDD, No. 36-1451).

Room-temperature PL measurements were performed using a 325 nm He–Cd laser excitation source (Kimmon Koha, Tokyo, Japan) coupled to a Renishaw spectrometer (Wotton-under-Edge, UK). Additional steady-state PL measurements were conducted using a Horiba Jobin Yvon Fluorolog-3 spectrofluorometer equipped with a Xe lamp and dual scanning monochromators. The PL decay characteristics were evaluated using a time-correlated single-photon counting technique with NanoLED excitation sources. PL spectra were corrected for instrumental response.

Surface elemental composition and chemical states were investigated by XPS using a Thermo Scientific Nexsa G2 system equipped with a monochromated, microfocused Al Kα X-ray source (hν = 1486.6 eV). The binding-energy scale was charge-corrected by referencing the adventitious carbon C 1s peak to 284.8 eV. Energy-dispersive X-ray spectroscopy (EDX) was performed using an EDX detector integrated with a Hitachi SU8230 cold-field-emission scanning electron microscope. The EDX spectrum was used as complementary qualitative evidence for the presence of Bi together with the XPS results.

Diffuse reflectance and absorption measurements were carried out using a Cary 5000 UV–Vis–NIR spectrophotometer (Agilent Technologies, USA) equipped with tungsten-halogen and deuterium light sources. Electron paramagnetic spectrometer (EPR) measurements were performed on the Bruker ELEXSYS-II E500 Continuous Wave EPR spectrometer operating in the X-band with the ELEXSYS Super High Sensitivity Probehead. The magnetic field was calibrated, and the sample was tuned prior to measurement. We ensured we were not operating in a power-saturating region for the room-temperature measurements.

CL measurements were performed by using an FEI Quattro environmental scanning electron microscope equipped with a Delmic SPARC CL detection module. CL spectra were collected by using an Andor Kymera 193 spectrograph coupled to an Andor Newton CCD detector. Measurements were conducted under a 250 mTorr water vapor environment to minimize the charging effects. An electron beam energy of 5 keV with beam currents ranging from 14 to 57 pA was used during the CL experiments.

### 2.3 RL Measurements

RL measurements were carried out under continuous X-ray excitation using a CMX003 X-ray generator. The emission spectra were recorded over the wavelength range of 200–800 nm using a 150 mm focal-length monochromator in both reflection and transmission geometries. The polychromatic X-ray spectra were generated at tube voltages of 35 and 45 kV, corresponding to nominal maximum photon energies of 35 and 45 keV, respectively. The initial Bi-assisted single-side and refresh-growth ZnO samples were measured under 35 kV X-ray tube voltage, whereas the double-sided structures were characterized using 45 kV X-ray tube voltage.

## 3 Results and Discussion

Figure 1 schematically illustrates the hydrothermal growth strategy, the PEI-assisted anisotropic ZnO NR formation mechanism, and the different scintillator architectures investigated in this study. During hydrothermal synthesis, Zn^2+^ and OH^–^ species supplied by zinc nitrate and HMTA gradually form Zn-containing hydroxide complexes in the supersaturated solution, followed by nucleation and subsequent crystal growth. The evolution of the precursor concentration can be qualitatively described by a LaMer-type growth model, where the supersaturation regime promotes burst nucleation, followed by a growth-dominated stage after the precursor depletion begins. In the presence of PEI, the growth behavior becomes strongly anisotropic because of preferential adsorption of PEI molecules on the nonpolar lateral facets of ZnO NRs. This suppresses lateral crystal growth while maintaining efficient incorporation of growth species on the polar (0001) top facet, thereby promoting preferential elongation along the *c*-axis direction and enabling the formation of vertically aligned high aspect ratio ZnO NR arrays.

**Figure 1:**
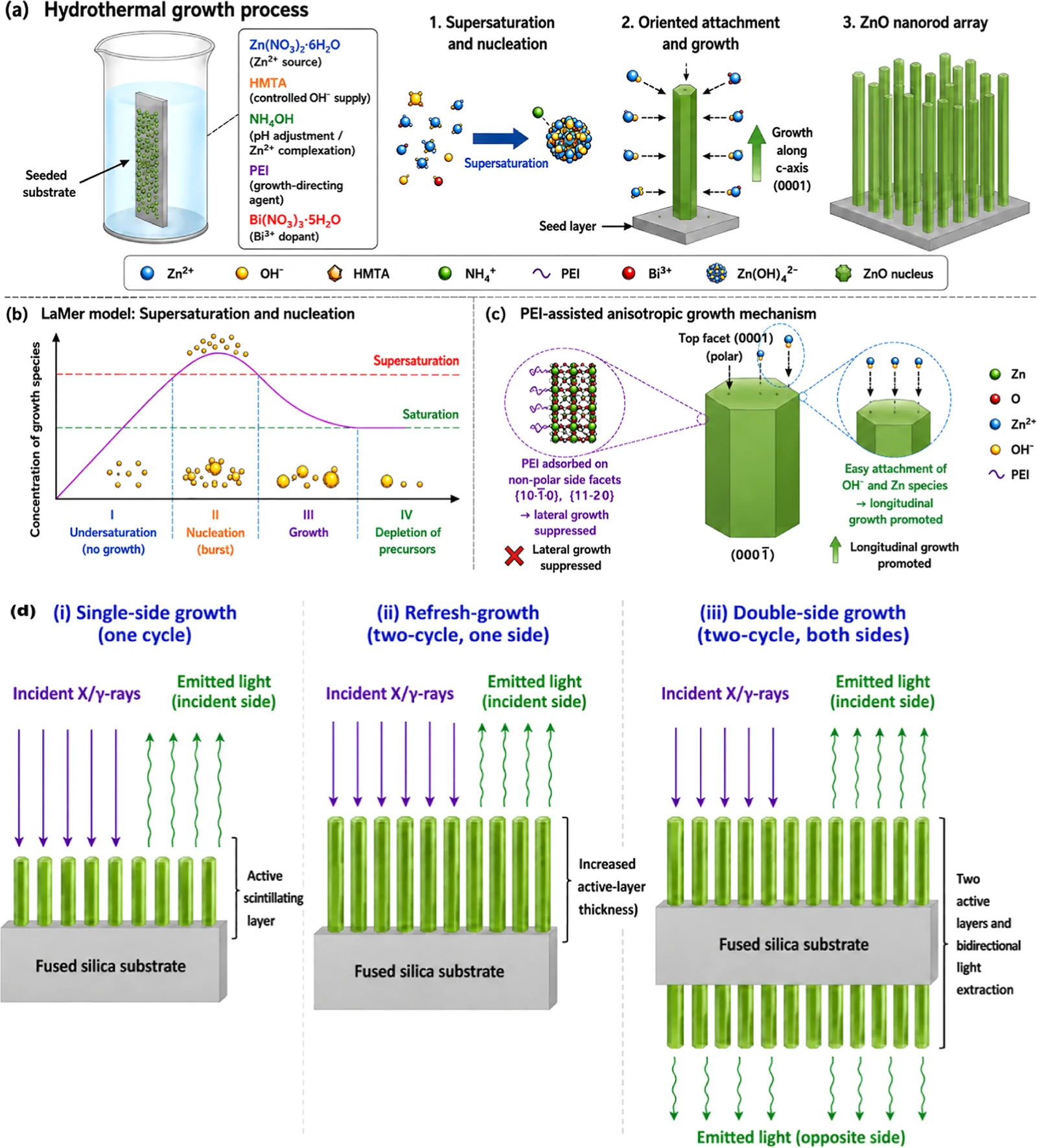
Schematic illustration of the growth process and the resulting ZnO nanorod (NR) architectures. (a) Hydrothermal growth process of the Bi-assisted ZnO NR arrays, illustrating precursor supersaturation and nucleation, oriented attachment and preferential growth along the *c*-axis, and formation of the NR array on the seeded substrate. (b) LaMer-type representation of the successive undersaturation, burst-nucleation, growth, and precursor-depletion stages. (c) Proposed PEI-assisted anisotropic growth mechanism, in which preferential PEI adsorption on the nonpolar side facets suppresses lateral growth while attachment of Zn and O species at the polar top facet promotes longitudinal growth. (d) Comparison of the scintillator architectures and their associated light-emission pathways: (i) single-side growth produced in one cycle, (ii) refresh growth produced through two successive cycles on the same side, and (iii) double-side growth produced through two growth cycles on opposite sides of the fused-silica substrate. The schematics are not drawn to scale.

Three different scintillator architectures were prepared to investigate the influence of geometry and effective scintillating thickness on the optical and scintillation behavior of the ZnO NR arrays, namely, single-side growth, refresh-growth, and double-sided growth structures. In the refresh-growth approach, the initially formed NR arrays were subjected to an additional hydrothermal growth cycle using a freshly prepared precursor solution in order to further increase the active scintillating layer thickness. In contrast, the double-sided architecture was achieved by sequentially growing ZnO NR arrays on both sides of the fused silica substrate, resulting in an increased effective interaction volume together with a bidirectional light extraction capability. Such architecture-dependent modifications are expected to influence the X-ray interaction depth, optical transport, and RL response of the ZnO NR scintillator structures.

The morphological properties of the Bi-assisted ZnO NR scintillator structures are presented in Figure 2. The low-magnification top-view SEM image shown in Figure 2a reveals highly uniform large-area coverage consisting of densely packed and vertically aligned ZnO NR arrays over the fused-silica substrate. The corresponding high-magnification inset further confirms the formation of well-defined hexagonal NR morphologies with relatively narrow diameter variations and a high surface coverage density. Such dense and vertically aligned architectures are advantageous for scintillator applications, since they may promote directional optical transport within the NR array geometry while maintaining a large effective scintillating volume.

**Figure 2:**
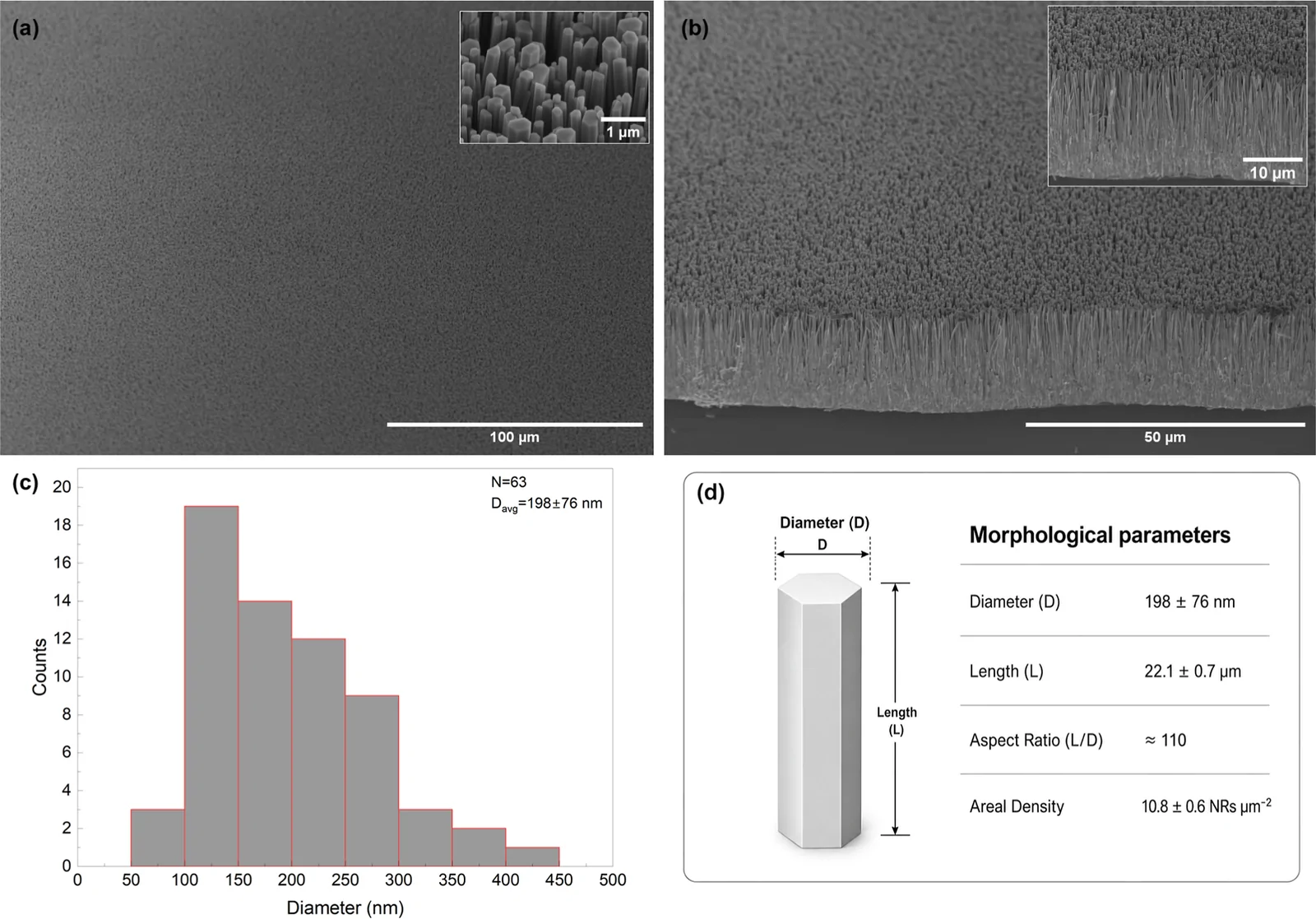
Morphological characterization of the (5%) Bi-assisted ZnO nanorod (NR) arrays. (a) Low-magnification top-view SEM image demonstrating the large-area uniformity and dense packing of the vertically aligned ZnO NR arrays, together with a high-magnification inset showing the hexagonal NR morphology. (b) Cross-sectional SEM image with inset revealing the vertically aligned high-aspect-ratio NR architecture. (c) Diameter distribution histogram obtained from individual ZnO NRs. (d) Summary of the extracted morphological parameters including average diameter, NR length, aspect ratio, and areal density.

The cross-sectional SEM image in Figure 2b demonstrates the formation of continuous high-aspect-ratio ZnO NR layers with relatively uniform vertical alignment throughout the entire thickness of the active scintillating structure. The inset image further illustrates the elongated NR morphology and dense rod packing obtained after the hydrothermal growth process. The average NR length extracted from multiple SEM regions was approximately 22.1 ± 0.7 μm, while the average diameter determined from statistical analysis of 63 individual NRs was 198 ± 76 nm (Figure 2c). The relatively broad diameter distribution may originate from local supersaturation fluctuations, secondary radial growth, and partial NR coalescence occurring during prolonged hydrothermal growth.

The extracted structural parameters summarized in Figure 2d indicate an average areal density of 10.8 ± 0.6 NRs μm^–2^ together with an aspect ratio approaching ∼110. Compared with the undoped ZnO NRs, the Bi-assisted samples exhibit shorter NR lengths, which may be attributed to Bi ions in the precursor solution imposing an additional thermodynamic barrier to ZnO nucleation and axial growth on the seed layer, thereby reducing the growth rate of primary nuclei, as reported previously for Bi-modified ZnO nanostructures.^41^ Interestingly, although previously reported undoped ZnO NR arrays exhibited larger absolute NR lengths,^4^ the comparatively reduced NR diameters obtained in the present Bi-assisted structures resulted in higher aspect ratio architectures. Such morphology evolution may be advantageous for scintillator architectures where directional optical transport and reduced lateral photon scattering are desirable. Additional SEM images of the undoped, refresh-grown, and double-sided ZnO NR architectures are provided in Figure S1. The undoped ZnO sample exhibited a comparable vertically aligned NR morphology, although the Bi-assisted arrays showed a higher aspect ratio due to their reduced average NR diameter. The refresh-grown Bi-assisted sample formed a substantially thicker single-side NR layer, confirming the effectiveness of the second hydrothermal growth cycle in increasing the active scintillating thickness. Representative cross-sectional SEM images collected from opposite sides of the double-sided architecture further support the successful formation of ZnO NR layers on both surfaces of the fused silica substrate.

The crystal structure and preferential orientation of the ZnO NR arrays were further investigated by XRD analysis, as shown in Figure 3. The stacked XRD patterns in Figure 3a show that all samples exhibit a dominant ZnO(002) reflection together with a weaker (004) reflection, confirming the preferential *c*-axis-oriented growth of the wurtzite ZnO NR arrays. The experimental diffraction peaks are in good agreement with the standard hexagonal wurtzite ZnO reference pattern (JCPDS No. 36-1451),^6^ and no clear secondary phase reflections associated with Bi-containing compounds are observed within the detection limit of the measurement. This indicates that the wurtzite ZnO crystal structure is largely preserved after low-to-moderate Bi incorporation and refresh growth.

**Figure 3:**
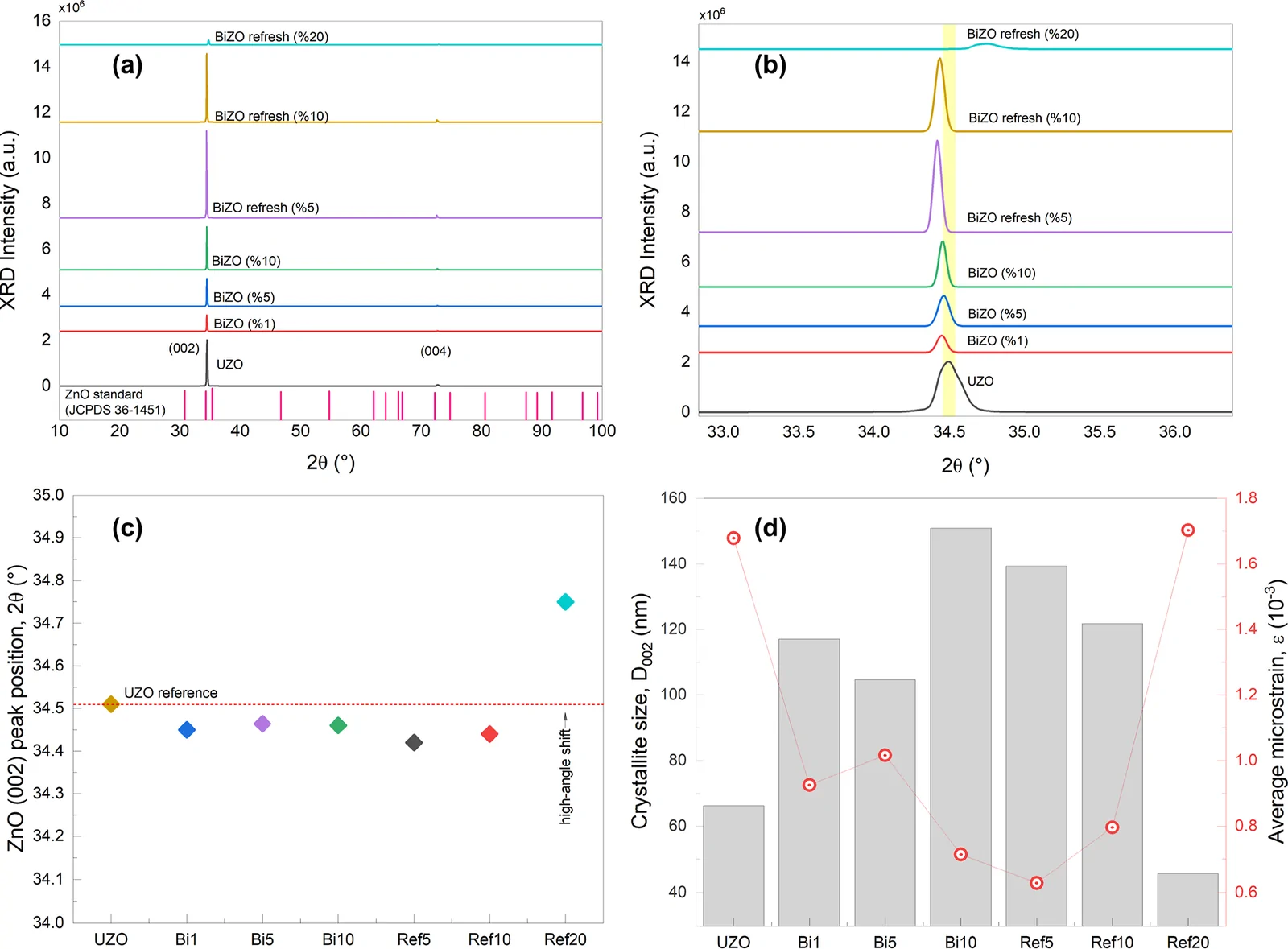
XRD analysis of undoped, Bi-assisted, and refresh-grown ZnO:Bi NR arrays. (a) Stacked XRD patterns together with the standard ZnO reference pattern (JCPDS 36-1451), showing the dominant *c*-axis-oriented ZnO reflections. (b) Enlarged view of the ZnO (002) reflection region highlighting the peak-shift behavior with Bi incorporation and refresh growth. (c) Variation of the ZnO (002) peak position extracted from peak fitting analysis. (d) Scherrer crystallite size, D_002_ (nm) calculated from the ZnO (002) reflection and average microstrain values as a function of sample type. Bi1, Bi5, and Bi10 denote nominal 1, 5, and 10% Bi-assisted ZnO NR arrays; ref 5, ref 10, and ref 20 denote refresh-grown ZnO:Bi samples.

The enlarged view of the ZnO(002) reflection region in Figure 3b reveals a clear peak-shift behavior as a function of Bi addition and growth conditions. Compared with the undoped ZnO sample, the Bi-assisted samples show a slight shift of the (002) peak toward lower diffraction angles, suggesting an increase in the corresponding interplanar spacing along the *c*-axis direction, as similarly reported elsewhere.^38,41^ Such a shift may originate from the Bi-related lattice perturbation, local strain relaxation, or defect redistribution within the ZnO NR structure. However, for the refresh-grown 20% ZnO:Bi sample, the (002) reflection exhibits a pronounced high-angle shift, as also summarized by the extracted peak position values shown in Figure 3c. Since this anomalous behavior is not equivalently reflected in the (004) peak analysis, it is more cautiously interpreted as evidence of nonuniform structural perturbation, local strain, or peak shape/texture effects under excessive nominal Bi loading rather than a simple uniform contraction of the ZnO lattice.

For the XRD analysis, the peak positions and full widths at half-maximum (fwhm) of the ZnO (002) and (004) reflections were obtained by peak fitting. The corresponding coherent crystallite sizes were estimated using the Scherrer equation (eq 1)­

1 $$D_{hkl}=K\lambda /\left(\beta_{hkl}\cos\theta_{hkl}\right)$$

where K = 0.90, λ = 1.54187 Å, β_hkl_ is the fitted fwhm, and θ_hkl_ is the Bragg angle. The structural parameters extracted from the peak analysis further support the evolution of crystal quality with Bi incorporation and refresh growth. As shown in Figure 3d, moderate Bi-assisted growth leads to an increase in the crystallite size calculated from the ZnO (002) reflection, accompanied by a reduction in the average microstrain. This trend indicates improved structural ordering along the *c*-axis direction and is consistent with the narrower diffraction peaks observed for the Bi-assisted samples. In particular, the moderate Bi and refresh-grown samples show larger crystallite sizes and lower microstrain compared with the undoped ZnO reference, suggesting that controlled Bi-assisted growth can improve the crystallographic quality of the vertically aligned ZnO NR arrays.

In contrast, the ref20 sample exhibits a marked decrease in crystallite size together with an increase in average microstrain. This behavior is consistent with the anomalously high-angle shift of the ZnO (002) reflection and suggests that excessive nominal Bi loading disrupts the structural ordering of the ZnO NR arrays. Therefore, while low-to-moderate Bi incorporation appears to preserve the wurtzite ZnO structure and improve the *c*-axis-oriented crystal quality, excessive Bi concentration likely introduces structural disorder, nonuniform strain, and defect-rich growth conditions.

Additional XRD-derived structural parameters are provided in Figure S2 and Table S1. The peak fitting parameters extracted from both the ZnO (002) and (004) reflections, including peak center position, fwhm, crystallite size, and microstrain, are summarized in Table S1. The complementary parameters shown in Figure S2, including the average c-lattice parameter, lattice-strain-related quantities, average microstrain, and A(002)/A(004)-based orientation metrics, further support the structural evolution discussed above. In particular, the (004)-derived parameters follow trends broadly consistent with those obtained from the (002) reflection, confirming that the observed improvement in structural ordering at low-to-moderate Bi contents is not limited to a single diffraction peak. The deviation observed for the ref 20 sample further supports the conclusion that excessive nominal Bi loading induces nonuniform structural perturbation rather than simple lattice expansion or contraction. Nevertheless, the present measurements do not uniquely distinguish substitutional or interstitial Bi incorporation from a possible surface- or interface-associated Bi fraction.

The optical response of the ZnO NR arrays was further investigated using diffuse reflectance spectroscopy and Kubelka–Munk analysis, as shown in Figure 4. The diffuse reflectance spectra reveal a strong morphology-dependent optical response across the UV–visible region. Compared with UZO, the Bi-assisted and refresh-grown ZnO NR arrays exhibit a broadened and red-shifted reflectance response, with the main reflectance maximum shifting toward the visible region. This behavior indicates that Bi incorporation and extended refresh growth modify the optical scattering and absorption characteristics of the long NR architectures. Since vertically aligned and high-aspect-ratio NR arrays can strongly affect the optical path length through multiple scattering and light trapping, the extracted optical transition energies should be interpreted as apparent values rather than as a direct measure of the intrinsic ZnO band gap.

**Figure 4:**
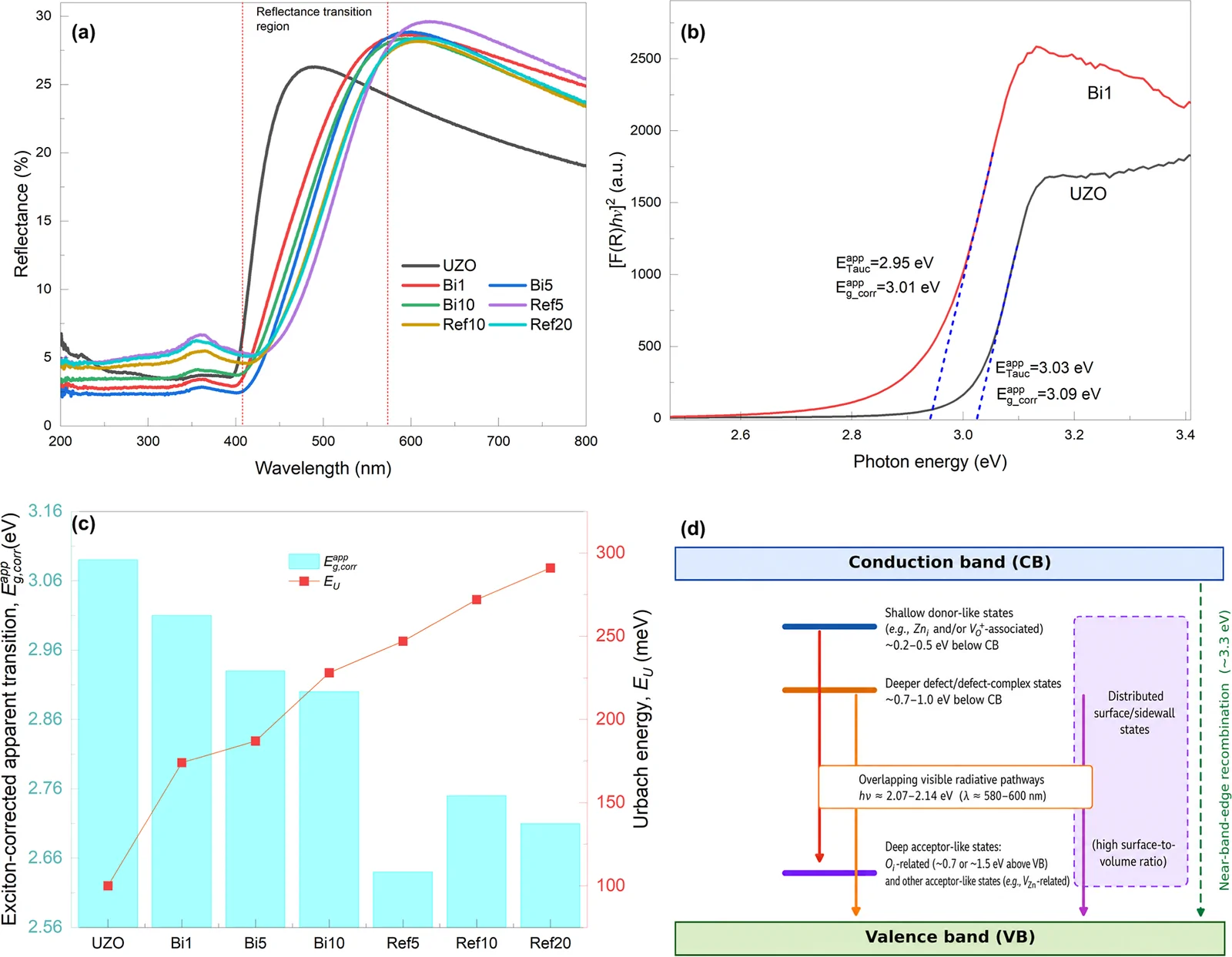
Optical response and defect-related band-tail analysis of ZnO nanorod arrays. (a) Diffuse reflectance spectra of UZO, Bi-assisted ZnO, and refresh-grown ZnO NR samples. (b) Representative Kubelka–Munk Tauc plots, [*F­(R)*hν][Bibr ref2] versus photon energy, showing the extraction of the lower-energy apparent optical transition *E*_*g*_^app^ and the corresponding exciton-corrected values. (c) Evolution of the exciton-corrected *E*_*g*_^app^ and Urbach energy *E*_*U*_ derived from diffuse reflectance data. (d) Qualitative energy-level scheme illustrating the near-band-edge transition and possible defect-mediated visible-emission pathways in the ZnO NR arrays. The broad emission at approximately 580–600 nm (2.07–2.14 eV) is represented as an overlapping radiative envelope that may involve shallow donor-like states, deeper defect/defect-complex states, *O*_*i*_-related deep acceptor-like states, other acceptor-like states including *V*_*Zn*_-related states, and distributed surface/sidewall states. The indicated state positions and transition pathways are approximate and are not intended to imply unique microscopic defect assignments.

To estimate the lower-energy optical transition associated with the long, visible-emission-dominant NR structures, Kubelka–Munk-transformed Tauc plots were constructed using [*F­(R)*hν][Bibr ref2] versus photon energy, where F­(*R*)=(1–*R*)^2^/2R. Representative plots for UZO and Bi1 are shown in Figure 4b. Their Tauc-extrapolated values of approximately 3.03 and 2.95 eV correspond to exciton-corrected apparent optical transition energies of approximately 3.09 and 3.01 eV, respectively. The corrected values remain apparent transition energies and are not interpreted as intrinsic ZnO band gaps. The decrease from UZO to Bi1 confirms that the apparent optical transition remains red-shifted after low-level Bi-assisted growth. This shift should not be regarded as a simple narrowing of the intrinsic ZnO band gap; rather, it is more reasonably attributed to enhanced defect-related sub-band absorption and band-tail states in the long ZnO NR architecture.^44,45^

The trend in Figure 4c further supports this interpretation. With increasing Bi incorporation and refresh growth, *E*_*g*_^app^ generally decreases, while the Urbach energy *E*_*U*_, extracted from the linear region of In­[*F­(R)*] versus photon energy, increases. This inverse relationship between *E*_*g*_^app^ and *E*_*U*_ suggests a progressive broadening of the absorption tail and an increased contribution from localized defect-related states. The refresh-grown samples, particularly ref 5–20 , show higher *E*_*U*_ values than UZO, indicating stronger optical disorder and enhanced band-tail absorption. This behavior is consistent with the structural results discussed above, where refresh growth and excessive nominal Bi loading modified the crystallite size, microstrain, and peak position of the ZnO NR arrays.

An energy-level scheme illustrating the proposed excitation, carrier-trapping, and radiative-recombination pathways is presented in Figure 4d. In addition to near-band-edge recombination, ZnO nanostructures may contain shallow donor-like states associated in the literature with Zn_*i*_- and/or V_O_^+^-, deeper defect/defect-complex states, O_*i*_-related deep acceptor-like states, other acceptor-like states including *V*_Zn_- related states, and distributed surface and sidewall states.^14,21,26,46,47^ *O*_*i*_ has frequently been discussed as a deep acceptor, with reported energetic positions of approximately 0.7 or 1.5 eV above the valence-band maximum.^48^ However, the energetic positions and relative contributions of these states are strongly dependent on synthesis conditions, stoichiometry, and nanostructure morphology; consequently, visible-emission bands in ZnO cannot generally be assigned uniquely from their spectral positions alone. The broad yellow–orange emission observed here at approximately 580–600 nm, corresponding to transition energies of about 2.07–2.14 eV, is therefore interpreted as the combined contribution of overlapping native-defect- and surface-associated recombination pathways rather than as emission from a single uniquely identified defect. Accordingly, the state positions and transition pathways illustrated in Figure 4d are qualitative and are not intended to imply unique microscopic defect assignments.

The high surface-to-volume ratio of the long ZnO NRs is expected to increase the contribution of distributed surface and sidewall states, consistent with the enhanced CL intensity frequently observed at the NR edges and sidewall regions. Bi-assisted hydrothermal growth may further modify the relative populations and local environments of these native and surface-related states through changes in nucleation, morphology, surface chemistry, and structural disorder. Similar synthesis- and concentration-dependent changes in visible luminescence and defect populations have been reported in Bi-containing ZnO systems.^38,40–42^ The present data, however, do not establish a discrete Bi-related radiative level; Bi is therefore treated as a modifier of the growth and defect environment rather than being assigned conclusively as the direct luminescent center.

Importantly, the large spectral separation between the UV band-edge absorption and the visible-emission region may also reduce self-absorption losses and thereby favor scintillation-light extraction from the long NR architectures. Complementary UV–Vis measurements acquired in direct-transmission geometry and recorded in absorbance mode are provided in Figure S3a,d, including spectra obtained from the two opposite orientations of the double-sided samples. Representative front- and back-side diffuse-reflectance spectra are shown in Figures S3e,f. The close agreement between the spectra recorded in the two orientations indicates that the optical response is largely insensitive to which NR-coated face is oriented toward the incident beam. Because the NR arrays scatter light, the recorded absorbance represents attenuation of the direct beam and may include scattering losses in addition to optical absorption.

The steady-state PL spectra of the Bi-assisted and refresh-grown ZnO NR arrays are shown in Figure 5. All samples exhibit a broad visible emission band centered at approximately 580–600 nm, whereas the near-band-edge emission around 380 nm remains weak. Yellow–orange emission in ZnO within the approximately 575–600 nm range is frequently associated with recombination involving *O*_*i*_-related deep acceptor states.^21,48,49^ By contrast, *V*_*O*_-related luminescence is more commonly reported in the green spectral region, approximately 485–530 nm.^50,51^ Nevertheless, the reported microscopic assignments vary with synthesis conditions, stoichiometry, morphology, impurities, and surface chemistry, and broad overlapping visible bands cannot generally be attributed to a single defect solely from their spectral positions.^14,25,46,47,52–54^ The present 580–600 nm emission is, therefore, consistent with a contribution from *O*_*i*_-related acceptor states. However, considering the breadth of the band and its enhanced CL intensity at NR edges and sidewalls, it is interpreted as an overlapping native-defect- and surface-associated emission envelope rather than being assigned exclusively to a single microscopic defect.

**Figure 5:**
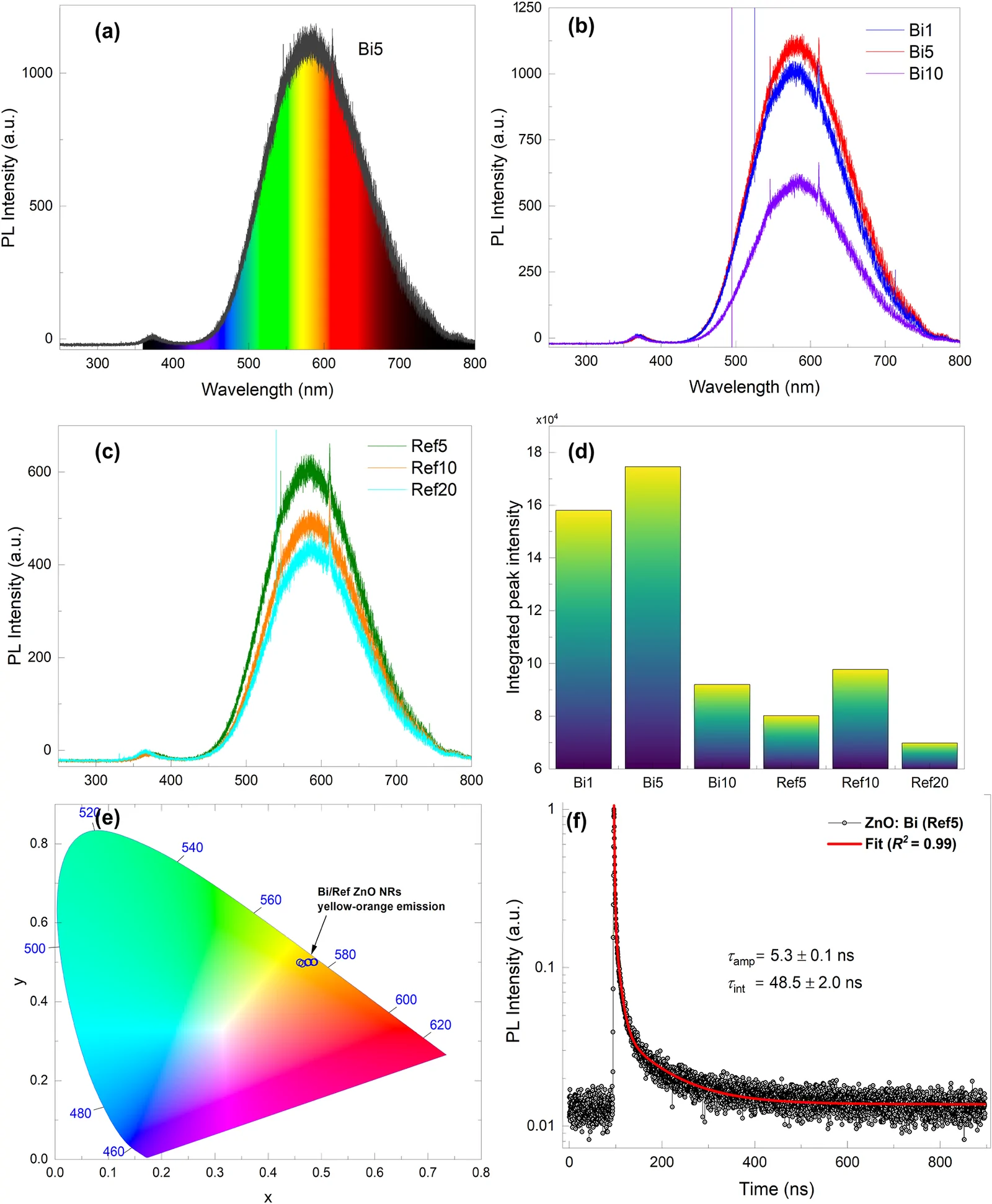
Photoluminescence response and recombination dynamics of ZnO:Bi NR arrays. (a) Representative visible PL emission from Bi5. PL spectra of (b) Bi-assisted ZnO NR samples and (c) refresh-grown ZnO:Bi NR samples. (d) Integrated (410–780 nm) visible PL intensity of the Bi-assisted and refresh-grown samples. (e) CIE 1931 chromaticity coordinates derived from the PL spectra, indicating yellow–orange emission. (f) Time-resolved PL decay of the representative refresh-grown ZnO:Bi sample fitted using a triexponential decay function.

The visible PL maximum also exhibits a slight red shift with increasing Bi-assisted growth conditions, shifting from ∼576 to ∼584 nm for Bi1–Bi10 and remaining around ∼585–587 nm for the refresh-grown samples (Figure 5b,c). Because the spectral profile remains broadly similar across the sample series, this modest shift is more consistent with changes in the relative populations and local environments of the contributing native and surface-related states than with the appearance of a new, discrete emission center. Bi-containing ZnO studies have likewise reported synthesis- and concentration-dependent changes in visible luminescence arising from modifications of native-defect populations, surface chemistry, and, in some systems, distinct Bi–O environments.^38,40–42^ Accordingly, the present PL data do not permit the broad yellow–orange emission to be assigned unambiguously either to a single native defect or to a direct Bi-related transition.

Among the Bi-assisted ZnO NR samples, Bi5 shows the strongest visible PL emission, followed by Bi1 and Bi10 (Figure 5b,d). This nonmonotonic trend suggests that moderate Bi-assisted growth favors radiative defect- and surface-associated recombination, whereas higher nominal Bi loading increases the relative contribution of nonradiative pathways. The reduced emission from Bi10 may be associated with increased structural/defect disorder and the formation of additional nonradiative recombination pathways. A similar nonmonotonic behavior is observed for the refresh-grown samples, where ref 10 exhibits the highest PL intensity among the refresh series, while ref 20 shows the weakest emission (Figure 5c,d). The reduced PL intensity of ref 20 is consistent with the XRD-derived structural perturbation and the higher Urbach energy obtained from diffuse reflectance analysis, suggesting that excessive nominal Bi loading and repeated growth can introduce optically active but partly nonradiative defect states.

The representative color-filled PL spectrum of Bi5 (Figure 5a) and the CIE 1931 chromaticity diagram (Figure 5e) further illustrate the yellow–orange emission character of the ZnO:Bi NR arrays. The CIE coordinates of the Bi-assisted and refresh-grown samples are clustered in the yellow–orange region, reflecting the similar spectral position of the broad visible emission band. This spectral clustering supports a common broad deep-level-emission envelope across the sample series. In agreement with the model in Figure 4d, the band is attributed to overlapping native-defect and surface-associated transitions, potentially including *O*_*i*_-related acceptor states, rather than to a sharp band-edge transition or a single uniquely identified defect.

Time-resolved PL measurements were further performed for the representative refresh-grown ZnO:Bi sample (ref 5) to evaluate the recombination dynamics of the visible-emission-dominant NR structure (Figure 5f). The decay profile monitored at 580 nm was described phenomenologically using a triexponential function, yielding characteristic times of τ_1_ = 1.4 ± 0.009 ns, τ_2_ = 10.5 ± 0.19 ns, and τ_3_ = 96.2 ± 2.7 ns (Figure S4c). The corresponding amplitude-weighted effective decay time was τ_amp_ ≈ 5.3 ± 0.1 ns, whereas the intensity-weighted decay time was τ_int_ ≈ 48.5 ± 2.0 ns because of the contribution of the extended decay tail. Thus, the approximately 5.3 ns value should not be interpreted as a single-component lifetime. The intensity-weighted lifetime is shorter than the previously reported value for undoped ZnO LNRs,^4^ indicating that Bi-assisted refresh growth can preserve relatively fast recombination dynamics despite the strong visible defect emission. The shortest fitted term is comparable to the temporal width of the measured instrument response function (IRF) and is, therefore, not interpreted as an independently resolved intrinsic lifetime or assigned to a specific transition. Nevertheless, the pronounced decay tail extends well beyond the IRF, confirming that the overall response is not instrument-limited. The fitted components are treated phenomenologically as characteristic contributions from superposed recombination, trapping, and detrapping pathways associated with the broad defect-mediated visible emission. Multiexponential functions have been widely used to describe ZnO emission dynamics involving superposed recombination, trapping, and detrapping pathways, including bi- and triexponential analyses of ZnO nanostructures.^4,55–57^ In contrast, the monoexponential response reported by Turtos et al. for ZnO concerned spectrally narrow near-band-edge emission in the 380–420 nm range and, therefore, represents a different emission regime from the broad defect-dominated yellow–orange emission monitored at 580 nm in the present study.^58^

Overall, the PL results support a balanced role of Bi incorporation and refresh growth: moderate Bi-assisted growth enhances the defect-related visible emission intensity, while excessive nominal Bi loading or repeated growth under Bi-rich conditions can increase disorder and nonradiative recombination. This trend agrees with the combined XRD and UV–vis results, where improved structural ordering was observed at moderate Bi levels, whereas Ref 20 exhibited structural perturbation, increased band-tail disorder, and reduced PL output. The Xe-lamp-based PLE spectra monitored at λ_em_ = 600 nm show strong UV/near-UV excitation bands, further supporting efficient excitation of the broad yellow–orange emission through the ZnO band-edge/near-band-edge absorption region (Figure S4a,b). Room-temperature continuous-wave electron paramagnetic resonance (CW-EPR) of the Bi-assisted ZnO NR sample shows a resonance at *g* ≈ 1.9595, consistent with the characteristic *g* ≈ 1.96 signal observed in our previous annealed ZnO NR-based systems^59^ and widely reported in ZnO nanostructures (Figure S 4d).^60,61^ This signal is commonly discussed in terms of shallow-donor-like or core/bulk-related centers, whereas resonances near *g* ≈ 2.00–2.005 are often associated with surface/shell-related paramagnetic defects.^23,61,62^ In the present Bi-assisted ZnO NRs, no detectable *g* ≈ 2.0048 signal was observed, although the sample exhibits dominant visible emission. This indicates that the visible defect-related emission does not directly correlate with EPR-active surface/shell paramagnetic centers detectable at g ≈ 2.0048 under room-temperature CW-EPR conditions, consistent with previous reports showing that visible PL in ZnO nanostructures does not necessarily exhibit a simple one-to-one correlation with EPR-active centers.^63^ Additional RT-TRPL measurements on representative ZnO:Bi NR samples show measurable nanosecond-scale decay dynamics with dominant fast components in the ∼2–12 ns range, further supporting the fast defect-mediated emission behavior of the NR arrays (Figure S4e–g). Time-resolved PL measurements recorded from one coated face of the double-sided Bi1, Bi5, and Bi10 arrays revealed that all fitted decay components progressively increased with increasing nominal Bi concentration (Figure S4e–g). In contrast, the amplitude-weighted average lifetime varied nonmonotonically within the relatively narrow range of 9.5–11.9 ns because the relative amplitudes, and consequently the weighting of the individual decay components, differed among the samples. These results indicate that increasing nominal Bi content modifies both the characteristic recombination times and the relative contributions of the optically probed decay pathways. The multiexponential components are interpreted phenomenologically and are not assigned uniquely to individual defect states.

To further elucidate the spatial origin of the visible emission, CL imaging and spectral mapping were performed on representative ZnO:Bi NR arrays (Figure 6). The SEM image confirms the formation of well-defined hexagonal top facets, while the corresponding CL image reveals a spatially heterogeneous emission contrast across the NR surface (Figure 6a,b). Notably, the CL intensity is not maximized at the center of the top facets; instead, stronger emission contrast is observed along the rod edges and sidewall regions, as similarly reported in previous studies in ZnO NRs.^29,32,64^ This behavior suggests that the broad visible luminescence is strongly influenced by surface- and sidewall-associated defect states rather than being uniformly generated throughout the NR volume.

**Figure 6:**
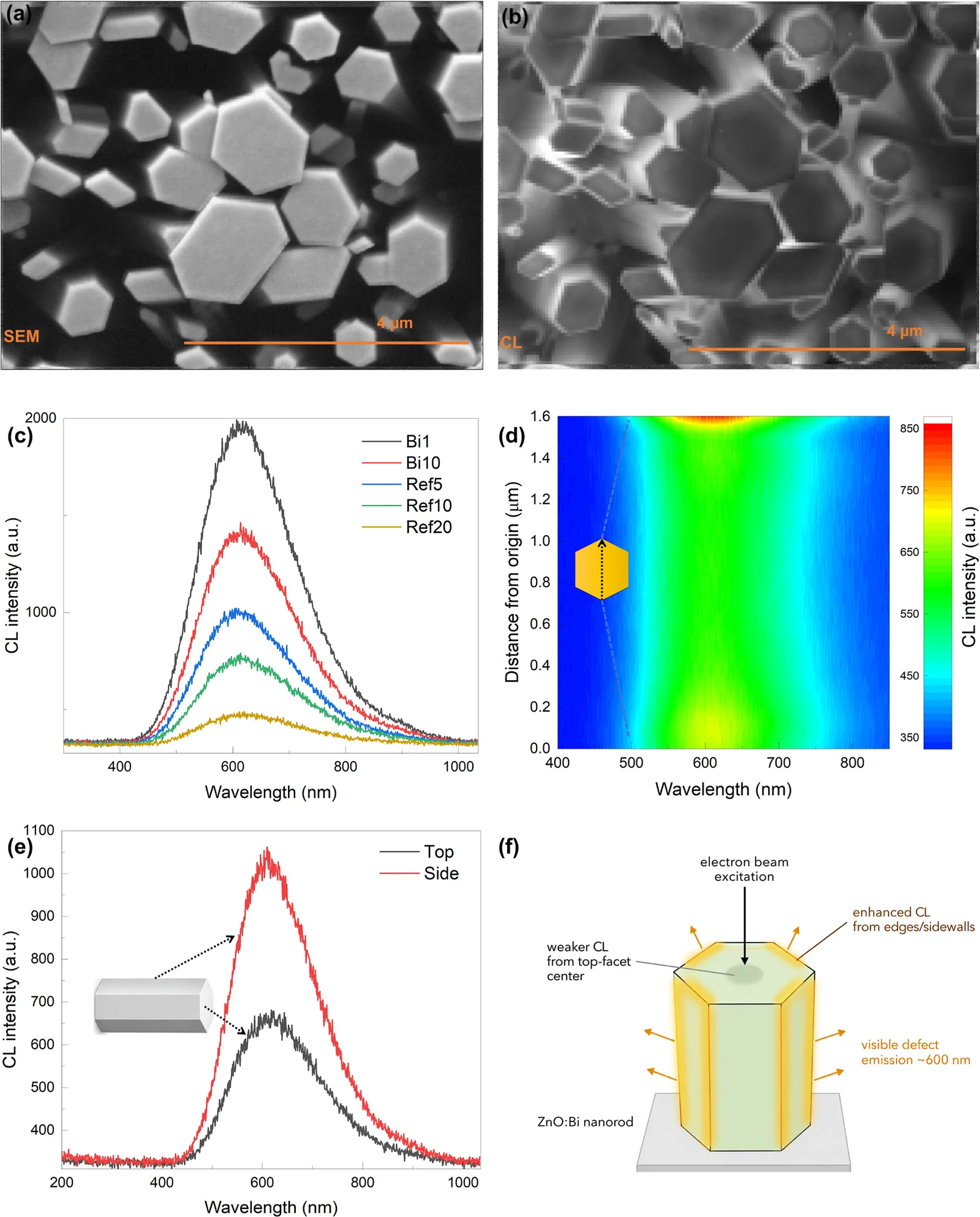
Cathodoluminescence imaging and spectral mapping of ZnO:Bi nanorod arrays. (a,b) Representative SEM and corresponding CL images of ZnO:Bi NRs. (c) CL spectra of selected Bi-assisted and refresh-grown samples, showing broad visible emission centered at ∼600 nm. (d) CL spectral line map collected across a hexagonal nanorod top facet, revealing stronger emission toward the rod edges. (e) Top-facet and sidewall CL spectra, showing enhanced sidewall-associated visible emission. (f) Schematic representation of the spatially heterogeneous CL response, where electron-beam excitation produces weaker emission from the top-facet center and stronger defect-mediated yellow–orange emission from edge/sidewall regions.

The CL spectra of the Bi-assisted and refresh-grown samples show a broad visible emission band centered at ∼600 nm (Figure 6c), in good agreement with the room-temperature PL spectra discussed above. This spectral consistency indicates that both optical excitation and electron-beam excitation predominantly activate the same defect-mediated yellow–orange emission pathway. However, the relative CL intensities vary significantly among the samples. In particular, ref 20 exhibits the weakest CL response while retaining the same broad visible emission profile. This behavior is consistent with the reduced PL intensity and increased optical disorder inferred from the Urbach energy analysis, suggesting that excessive nominal Bi loading during refresh growth introduces additional structural/defect disorder and nonradiative recombination pathways. Therefore, while moderate Bi-assisted growth promotes visible defect-related luminescence, excessive Bi-rich refresh growth appears to reduce the radiative efficiency of the NR arrays. Consistently, representative SEM/CL image pairs acquired under identical display conditions show that the refresh-grown samples, particularly those reported in ref 20, exhibit a more diffuse and weaker CL contrast compared with the Bi-assisted samples (Figure S5). This observation agrees with the lower CL spectral intensity of ref 20 in Figure 6c and further supports the view that excessive nominal Bi loading during refresh growth promotes disorder/nonradiative pathways that suppress visible radiative output. The CL spectral line map collected across the hexagonal top facet further confirms that the visible emission band extends across the probed NR region, with enhanced intensity toward the edge regions (Figure 6d). This edge-enhanced CL response is consistent with previous DFT-based studies showing that oxygen-vacancy-related defect formation is energetically more favorable near ZnO NR boundaries/edges, resulting in a higher defect density in these regions.^33^ Although oxygen-vacancy-related defect formation may be favored near ZnO NR boundaries, this spatial variation does not by itself identify the microscopic center responsible for the 580–600 nm emission band. This spatial trend is consistent with the top/side CL comparison shown in Figure 6e, where the sidewall region exhibits a stronger CL response than the top-facet region.

The enhanced sidewall/edge emission is consistent with the high surface-to-volume ratio of the NR architecture and a larger contribution from distributed oxygen-related and surface-associated localized states at the exposed sidewall facets. Local morphology, surface inclination, electron-beam interaction volume, and collection geometry may also contribute to the observed contrast; therefore, the CL intensity distribution is interpreted as a combined effect of defect distribution and NR geometry. The schematic in Figure 6f summarizes this spatial emission picture: electron-beam excitation produces weaker CL from the top-facet center but stronger defect-mediated visible emission from the edge/sidewall regions. Overall, the CL results support the PL and UV–vis analyses, confirming that the yellow–orange emission in ZnO:Bi NR arrays is defect-mediated and spatially enhanced at sidewall/edge regions of the NR architecture. XPS and EDX analyses of a representative ZnO:Bi NR sample further support the presence of Bi species associated with the ZnO NR arrays and reveal surface/defect-related oxygen contributions in the O 1s spectrum (Figure S6).

X-ray-excited RL provides a direct and application-relevant assessment of scintillator performance, since X-rays generate carriers through highly penetrating ionizing interactions rather than surface-limited optical excitation. The resulting emission depends on energy deposition, carrier transport, trapping, radiative recombination, and light extraction, and is, therefore, sensitive to the composition, morphology, crystallinity, and defect landscape of the scintillator.^65^ Therefore, RL measurements were further performed under 45 kV X-ray tube voltage to evaluate the scintillation response of the double-sided ZnO:Bi NR arrays. The reflection and transmission measurement configurations used for these measurements are schematically illustrated in Figure 7. In the reflection configuration, the X-ray beam was incident at approximately 45° to the sample surface, and the RL signal was collected from the same side along an axis perpendicular to the incident beam. In the transmission configuration, the X-ray source, sample, and monochromator were aligned along the same central axis, with the RL signal collected from the side opposite to X-ray incidence. As shown in Figure 8a,b, all samples exhibit a broad visible RL band centered at ∼600 nm in both reflection and transmission geometries. The spectral position and broad band shape are consistent with the yellow–orange emission observed in the PL and CL measurements, indicating that the same defect-mediated visible recombination channel is activated under ionizing radiation. The front- and back-side measurements show similar spectral profiles, confirming that both NR-coated surfaces are optically active and contribute to the X-ray-induced emission.

**Figure 7:**
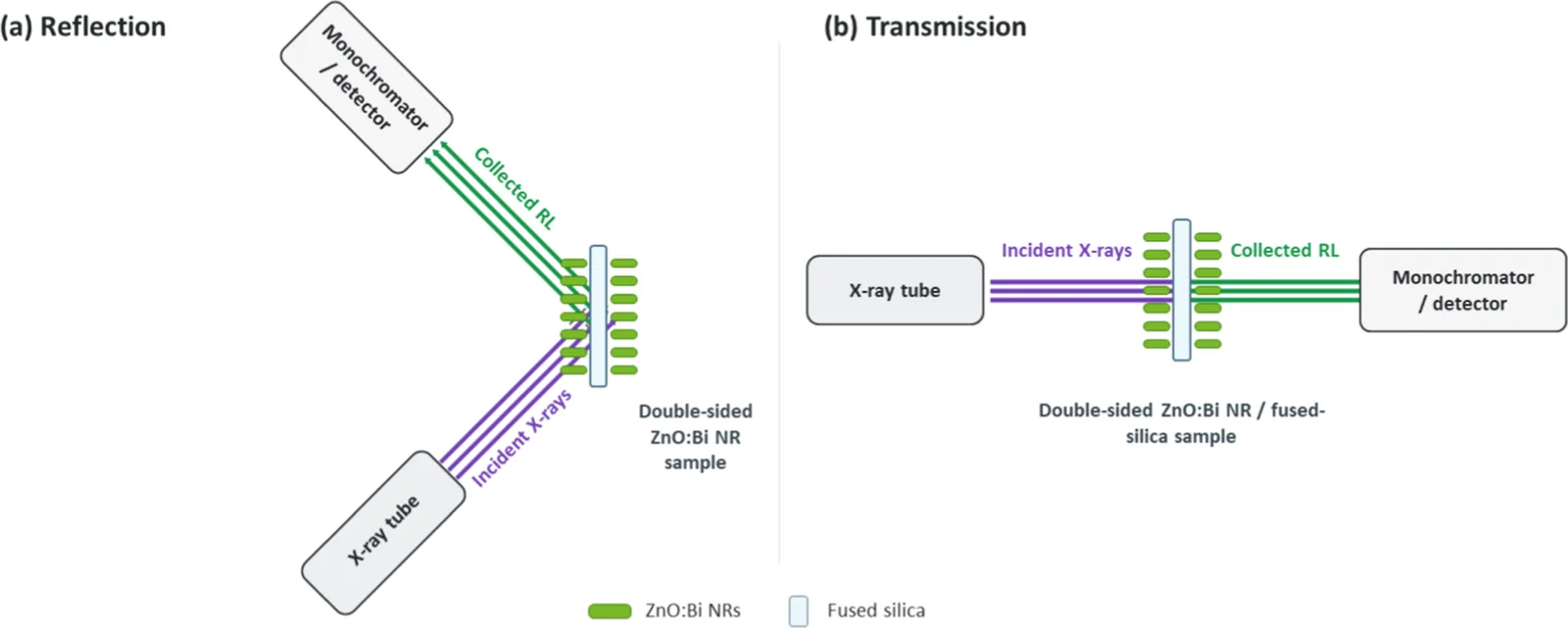
Schematic illustration of the RL measurement configurations used for the double-sided ZnO:Bi NR scintillators. (a) Reflection geometry, in which the X-ray beam was incident at approximately 45° to the sample surface and the RL signal was collected from the same side along an axis perpendicular to the incident beam. (b) Transmission geometry, in which the X-ray source, sample, and monochromator were aligned along the same central axis and the RL signal was collected from the side opposite to X-ray incidence. The schematic is not drawn to scale.

**Figure 8:**
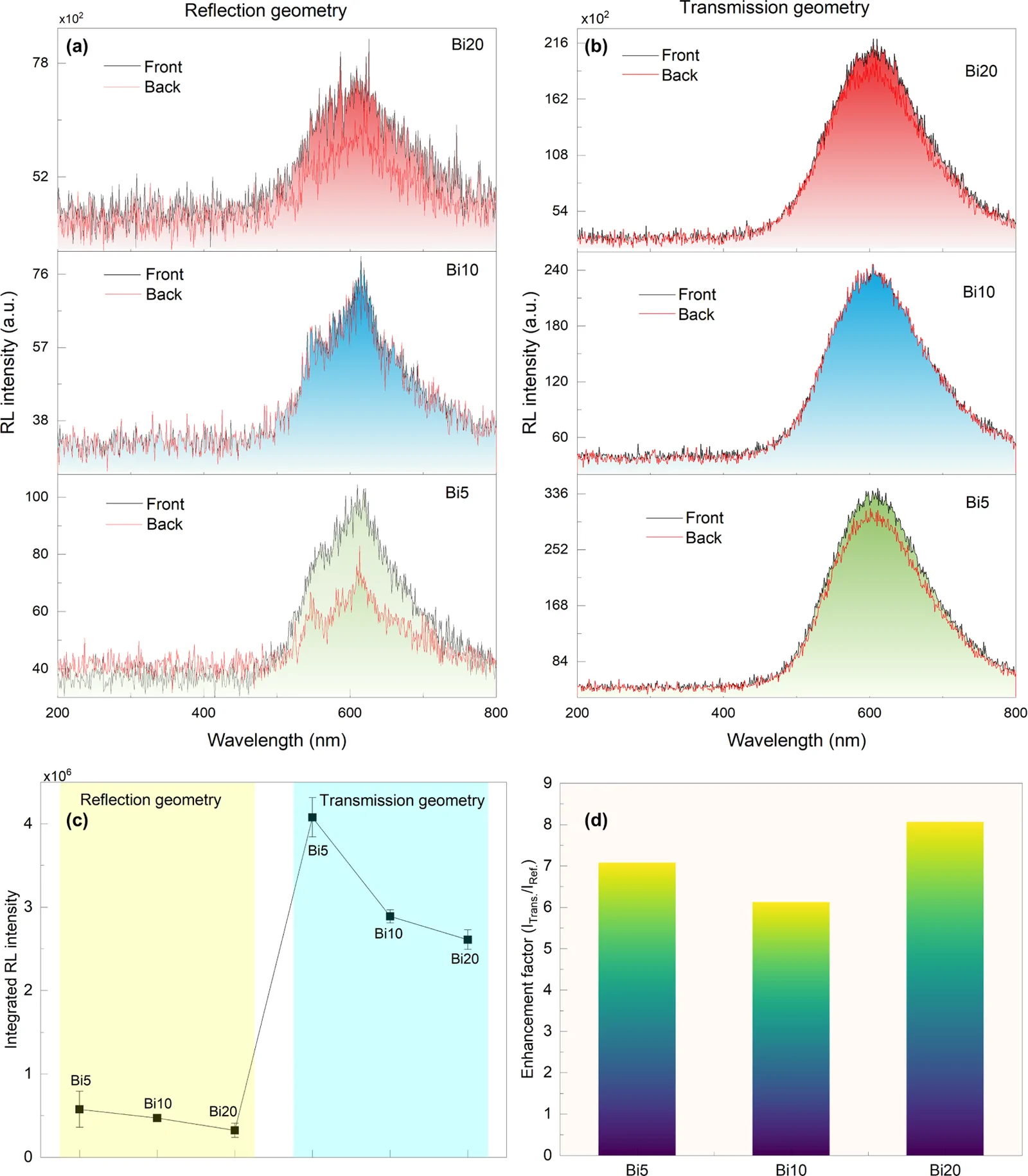
Radioluminescence response of double-sided ZnO:Bi NR arrays under 45 kV X-ray tube voltage. (a) Reflection-mode and (b) transmission-mode RL spectra collected from the front and back sides of Bi5, Bi10, and Bi20 samples (the *y*-axis is scaled by 10^2^ counts). All samples exhibit a broad visible emission band centered at ∼600 nm, consistent with defect-mediated yellow–orange emission. (c) Integrated RL intensities calculated from background-corrected spectra over the 450–800 nm range, showing a substantial enhancement in transmission geometry compared with reflection geometry. Error bars represent the variation between front- and back-side measurements. (d) Enhancement factors calculated as the ratio of integrated transmission to reflection RL intensity, *I*_trans*.*_ */I*_refl._.

A pronounced difference is observed between the two collection geometries. The RL intensity collected in transmission geometry is substantially higher than that measured in reflection geometry for all double-sided samples. Because the same double-sided samples and, therefore, the same active-material volume were used in both measurement configurations, the higher transmission-mode signal cannot be attributed simply to an increase in scintillator thickness. Instead, the result demonstrates a pronounced dependence of the detected RL intensity on the relative excitation, optical-transport, substrate-transmission, and collection geometries. The present measurements do not allow the individual contributions of these factors to be quantitatively separated. To quantify this behavior, integrated RL intensities were calculated from the background-corrected spectra over the 450–800 nm range (Figure 8c). The integrated spectra confirm a higher detected RL intensity in transmission than in reflection geometry under the applied measurement conditions. Among the investigated samples, Bi5 shows the highest measured transmission RL intensity, followed by Bi10 and Bi20, suggesting that moderate Bi-assisted growth provides the most favorable balance between visible-emitting defect states, NR morphology, and radiative efficiency. In contrast, higher nominal Bi loading does not further improve the RL output, likely due to increased structural/defect disorder and associated nonradiative loss pathways.

The enhancement factor, defined as *I*_trans._ */I*_refl_, remains high for all samples, with values of approximately 6–8 (Figure 8d). Although Bi5 exhibits the highest measured transmission RL intensity, the enhancement factor varies depending on the relative reflection intensity of each sample. Although the same double-sided sample and nominal X-ray tube settings were used, the change in measurement geometry alters both the X-ray incidence and the optical collection pathway. Therefore, the substantially higher RL intensity detected in transmission should not be interpreted as a corresponding increase in the intrinsic scintillation yield. Instead, it reflects the geometry-dependent probability that the generated photons reach the collection optics. A simplified escape-cone calculation provides a first-order, semiquantitative rationale for more efficient substrate-directed optical coupling. At the 580–600 nm emission wavelengths, the reported room-temperature refractive indices are approximately *n*_*o*_ ≈ 2.00–2.01 and *n*_*e*_ ≈ 2.02 for bulk crystalline ZnO^66^ and *n* ≈ 1.458–1.459 for fused silica.^67^ Using these values, the critical angle is approximately 30° at a ZnO–air interface and 46°–47° at a ZnO–silica interface (eqs 2 and 3)­

2 $$\theta_{c,ZnO/air}=\sin^{-1}\left(\frac{n_{air}}{n_{ZnO}}\right)\approx 30^{{}^{\circ}}$$

3 $$\theta_{c,ZnO/silica}=\sin^{-1}\left(\frac{n_{silica}}{n_{ZnO}}\right)\approx 46-47^{{}^{\circ}}$$

For ideal isotropic emission incident on a planar interface, the geometrical fraction contained within an escape cone scales as 1 – cos θ_*c*_. The corresponding escape-cone fraction toward fused silica is, therefore, approximately (eq 4)­

4 $$\frac{1-\cos\theta_{c,ZnO/silica}}{1-\cos\theta_{c,ZnO/air}}\approx 2.35$$

times that toward air. This calculation uses bulk-crystal refractive indices to describe local coupling from an individual ZnO NR into the fused-silica substrate; these values should not be interpreted as the effective refractive index of the entire porous NR array. The effective optical response of a porous ZnO NR array depends on its morphology and ZnO filling fraction and may, therefore, differ appreciably from that of bulk ZnO.^68^ This simplified estimate supports preferential substrate-directed coupling but does not constitute a quantitative model of light extraction from the NR architecture or independently account for the experimentally observed 6–8-fold difference. NR-mediated scattering and axial guiding, multiple interface interactions, differences in optical path and attenuation, and the finite collection solid angle of the experimental system may contribute collectively. The measured enhancement is, therefore, assigned to a system-level increase in detected transmission-mode RL specific to the present double-sided architecture and measurement arrangement, rather than to an equivalent enhancement in intrinsic scintillation light yield. Quantitative separation of the individual optical contributions would require angle-resolved optical measurements or detailed electromagnetic or ray-tracing simulations.

Overall, these results demonstrate a pronounced dependence of the detected RL signal on the collection geometry in the double-sided ZnO:Bi NR architecture, supporting its further investigation for thin, transparent scintillator configurations. The present RL measurements were designed to provide internally consistent comparisons among the investigated compositions, architectures, and measurement geometries. Because the system was not calibrated for absolute photon yield determination and no reference scintillator was measured under identical conditions, the detected intensities cannot be converted reliably into photons/MeV or energy conversion efficiency. The reported enhancement factors should, therefore, be interpreted as comparative changes in the integrated detected RL signal rather than as changes in the intrinsic scintillation yield. Future calibrated measurements against established reference scintillators would enable the absolute device-level performance of these architectures to be assessed.

Additional 35 kV RL measurements on single-sided ZnO and refresh-grown ZnO NR samples also show broad yellow–orange emission centered at ∼600 nm, confirming that the same defect-mediated visible recombination channel is activated under X-ray excitation (Figure S7a). Cross-sectional SEM analysis showed that the representative refresh-grown ref 5 array exceeded 50 μm in length, whereas the one-sided Bi-containing arrays were approximately 25 μm long, demonstrating a more than 2-fold increase in NR-layer length after the additional growth step (Figure 1b). Consistent with this structural difference, the refresh-grown samples generally exhibited higher detected RL intensities than the one-sided samples under the identical 35 kV tube voltage and collection conditions shown in Figure S7a. This group-level trend supports a positive association between the longer NR architecture and enhanced detected RL, plausibly through the increased amount of X-ray-interacting ZnO along the growth direction. The closely grouped RL responses within each growth series further indicate that the growth architecture exerts a more pronounced influence on the detected RL than the variation in nominal Bi concentration over the investigated range. Because cross-sectional SEM data for the refresh-grown series are represented by ref 5, the observed enhancement is not assigned exclusively to NR length; concomitant changes in NR morphology, defect-mediated emission, crystallinity, nonradiative recombination, and light collection may also contribute. The calculated X-ray mean free paths/attenuation lengths further indicate that, in the 35–45 keV range, X-rays can penetrate the fused-silica substrate and interact with the NR layers on both sides (Figure S7b), confirming that both layers are accessible under the applied excitation conditions. The NIST X-ray mass attenuation coefficients data were used to calculate the X-ray mean free paths.^69^ The one-sided and refresh-grown series in Figure S7a were evaluated at 35 kV, whereas the double-sided samples were measured at 45 kV. The double-sided samples were evaluated at 45 kV because the 35 kV condition did not provide a sufficiently reliable signal for this data set. Accordingly, absolute RL intensities were compared only within data sets acquired under identical excitation and collection conditions. For the double-sided architecture, the principal quantitative comparison is the 6–8-fold difference between the transmission and reflection detection geometries measured on the same sample under identical 45 kV conditions. The operational stability of the representative double-sided ZnO:Bi NR scintillator was further evaluated under continuous X-ray irradiation. As shown in Figure S8, the emission-band shape and peak position remained essentially unchanged over 120 min, while the integrated RL intensity retained approximately 96%–97% of its initial value. These results indicate no substantial degradation of the RL response under the applied irradiation conditions. The representative PLE/PL spectra further show efficient near-UV excitation and broad visible emission in the yellow–orange spectral region, consistent with the RL response (Figure S7c). In addition, the absorption/RL comparison for Bi5 shows that the X-ray-induced yellow–orange emission is spectrally separated from the strong near-band-edge absorption region, which can reduce reabsorption losses and favor efficient visible-light extraction (Figure S7d).

To place the present results within the broader context of ZnO-based scintillators and nanostructured X-ray-conversion systems, representative previous studies are compared in Table 1. As summarized in Table 1, ZnO-based scintillator studies vary considerably in excitation energy, material dimensions, emission mechanism, collection geometry, and temporal characterization method, precluding a direct quantitative comparison of reported luminescence intensities and decay values. The present work is distinguished by combining Bi-assisted visible-emitting ZnO NRs with a double-sided, optically transparent architecture and by directly comparing reflection and transmission collection geometries. The observed 6–8-fold increase, therefore, represents an enhancement in the detected transmission-mode RL relative to reflection for the same samples and measurement system, rather than an equivalent increase in intrinsic scintillation light yield.

**Table 1:** Comparison of Representative ZnO-Based Scintillators and Nanostructured X-Ray-Conversion Systems

study	material/architecture	preparation	radiation/measurement	emission	temporal response^a^	main reported outcome
Bourret-Courchesne et al. (2009)^70^	polycrystalline ZnO:Ga powder	high-temperature solid-state reaction followed by H_2_ reduction	X-ray-excited luminescence	near-band-edge UV, ∼390 nm	∼0.8 ns, luminescence decay	intense ultrafast UV response obtained under optimized oxygen-rich and H-treated conditions
Armelao et al. (2010)^71^	ZnO nano- and microstructures	flow-deposition synthesis	synchrotron soft-X-ray XEOL and TR-XEOL	UV band-edge and visible defect bands	TR-XEOL; multicomponent, morphology-dependent	demonstrated strong effects of morphology, crystallinity, and surface/bulk defects on X-ray-excited emission dynamics
Kobayashi et al. (2015)^72^	vertically aligned ZnO nanowire screen	electrodeposition	20 keV X-ray imaging	UV–visible emission	NR	nanowire screen produced ∼10.6% higher image intensity than a ZnO film and improved image sharpness
Li et al. (2016)^73^	Ga- and In-doped ZnO micro-/nanorod arrays	hydrothermal growth	X-ray-excited luminescence	broad visible emission, ∼450–750 nm	NR	In-doped arrays exhibited the strongest XEL; doping and morphology controlled the visible response
Xu et al. (2021)^7^	vertically aligned ZnO nanowire screen, ∼2 μm thick	hydrothermal growth	30 keV monoenergetic X-ray imaging	UV–visible emission	NR	achieved 9.8 lp mm^–1^ (∼51 μm line width) spatial resolution through nanowire-guided suppression of lateral light spreading
Chen et al. (2024)^74^	double-layer ZnO:Sc nanorod scintillator	hydrothermal growth	X-ray imaging	near-band-edge UV emission	<1 ns, TRPL	double-layer architecture increased the active material while retaining ultrafast UV-emission dynamics
Kurudirek et al. (2024)^4^	vertically aligned ultralong ZnO NRs, ∼50 μm	hydrothermal growth	30 keV XEL, reflection geometry	visible emission centered at ∼580–600 nm	τ_1_ = 2.5 ns and τ_2_ = 73.2 ns, TRPL	ultralong NRs showed substantially stronger XEL than short NRs because of greater interaction thickness and visible-emission dominance
Janbazi et al. (2026)^75^	ZnO:Ga microrod array, ∼1.36 μm long	hydrothermal growth		Strong near-band-edge and weak visible emission	∼10 ns PL component; ∼ 15 ns detector-pulse rise time	demonstrated X-ray/α sensitivity and fast electrical output in a coupled detector configuration
this work	Bi-assisted double-sided ZnO NR arrays on fused silica	sequential hydrothermal growth on both substrate surfaces	45 kV RL; reflection and transmission geometries	Broad yellow–orange RL centered at ∼600 nm	τamp ≈ 5.3 ns, TRPL^b^	transmission-mode detected RL was ∼ 6–8 times that in reflection; Bi5 produced the highest measured transmission-mode signal

a The reported temporal values were obtained by using different methods and should not be interpreted as directly comparable intrinsic scintillation decay constants.

b The present temporal response was measured by TRPL for the representative refresh-grown ref 5 sample, rather than by time-resolved X-ray excitation.

NR: not reported.

No absolute scintillation light yield in photons/MeV or direct comparison with a standard scintillator under identical measurement conditions was reported for the ZnO NR studies summarized here, including the present work.

## 4 Conclusions

In summary, Bi-assisted ZnO NR arrays were developed as thin, transparent, and defect-engineered scintillator architectures for X-ray detection. The hydrothermal growth strategy enabled dense, vertically aligned ZnO:Bi NR arrays on fused-silica substrates, including double-sided configurations designed to increase the available scintillating material while preserving optical transparency. Structural and optical analyses confirmed the preferential wurtzite ZnO orientation and showed that the Bi-assisted arrays retain a visible-emission-dominant response characterized by broad yellow–orange emission at ∼580–600 nm and weak near-band-edge emission. The combined PL, PLE, CL-mapping, and O 1s XPS results support an interpretation in which this broad emission arises from overlapping defect-mediated recombination pathways involving native oxygen-related states and distributed surface/sidewall states.

Time-resolved PL measurements revealed fast visible-emission dynamics, with an amplitude-weighted average lifetime of ∼5.3 ns, supporting the potential of these defect-engineered ZnO NR arrays for fast scintillator applications. The investigated structures combine high-aspect-ratio NR growth, spectrally separated near-band-edge absorption and visible emission, and a double-sided active-material geometry. Under a 45 kV X-ray condition, the double-sided ZnO:Bi NR arrays exhibited broad RL emission near ∼600 nm in both reflection and transmission geometries. For the same double-sided samples, the integrated detected RL intensity was approximately 6–8 times higher in transmission than in reflection geometry, demonstrating a pronounced geometry dependence without implying the corresponding increase in intrinsic scintillation yield. The present measurements, however, do not permit the individual contributions of X-ray energy deposition, optical transport, substrate transmission, self-absorption, and collection geometry to be quantitatively separated. Among the investigated samples, ZnO:Bi (5%) exhibited the highest measured transmission-mode RL signal, suggesting that moderate Bi-assisted growth provides a favorable combination of NR architecture and defect-mediated radiative response, whereas higher nominal Bi loading may increase the relative contribution of nonradiative pathways.

Overall, this work demonstrates a distinctive integration of Bi-assisted growth modulation with double-sided, substrate-supported ZnO NR arrays for X-ray scintillation. The novelty lies in this material–architecture combination and its experimentally demonstrated geometry-dependent RL response, rather than in the proposal of a fundamentally new intrinsic scintillation mechanism. These findings establish double-sided ZnO:Bi NR arrays as a promising platform for the further development of thin, transparent, and fast scintillator screens.

## Supplementary Material



## Notes

Additional SEM characterization of undoped, refresh-grown, and double-sided ZnO NR architectures, low-magnification top-view SEM image of undoped ZnO NR arrays with an inset showing the corresponding cross-sectional morphology, cross-sectional SEM image of the Bi-assisted refresh-grown ZnO NR layer, showing the formation of an extended high-aspect-ratio nanorod architecture, representative cross-sectional SEM images collected from opposite sides of the Bi-assisted double-sided ZnO NR architecture, supporting the formation of ZnO NR layers on both sides of the fused silica substrate, peak fitting parameters and calculated structural quantities extracted from the ZnO (002) and (004) reflections, including peak center position, fwhm, crystallite size, and microstrain, additional XRD-derived structural parameters of Bi-assisted ZnO NR arrays, lattice strain-related parameter, average c-lattice parameter, Δ*c*, average microstrain, A(002)/A(004) ratio, and normalized A(002)/A(004) ratio for undoped ZnO and Bi-assisted ZnO NR arrays, corresponding parameters for the refresh-grown Bi-assisted ZnO NR arrays, supplementary UV–vis optical characterization of ZnO NR arrays, absorbance spectra of UZO, Bi-assisted ZnO, and refresh-grown ZnO NR samples, front- and back-side absorbance spectra of double-sided Bi1, Bi5, and Bi10 samples, front- and back-side diffuse reflectance spectra of representative double-sided Bi1 and Bi10 samples, showing comparable optical response from both sides of the substrate, additional optical and EPR characterization of ZnO:Bi NR arrays, PLE spectra recorded by scanning the excitation wavelength while monitoring the emission at λ_em_ = 600 nm, emission spectra recorded under λ_exc_ = 360 nm excitation, time-resolved PL decay curve of the representative Ref5 ZnO NR sample and the corresponding IRF, the Ref5 decay exhibits a pronounced tail extending well beyond the measured IRF, confirming that the overall response is not instrument-limited, room-temperature CW-EPR spectrum of the Bi-assisted ZnO NR sample, additional RT-TRPL decay curves and three-component exponential fits for representative ZnO:Bi NR samples, showing measurable nanosecond-scale decay dynamics with dominant fast components in the ∼2–12 ns range and amplitude-weighted average lifetimes of approximately 9.5–11.9 ns, representative SEM images and corresponding CL images of selected Bi-assisted and refresh-grown ZnO:Bi nanorod arrays, XPS and EDX characterization of a representative ZnO:Bi nanorod sample, XPS survey spectrum and high-resolution Zn 2p, O 1s, and Bi 4f spectra, EDX spectrum of the same representative ZnO:Bi sample, showing Bi-related peaks together with Zn and O signals, supplementary optical emission, radioluminescence, and attenuation analysis of ZnO:Bi NR arrays, RL spectra of single-sided ZnO:Bi and refresh-grown ZnO NR samples recorded under 35 kV X-ray tube voltage, showing broad yellow–orange emission centered at ∼ 600 nm, calculated X-ray mean free path/attenuation length of SiO_2_ and ZnO as a function of photon energy, supporting X-ray penetration through the fused-silica substrate in the 35–45 keV range, representative PLE and PL emission spectra of a ZnO:Bi NR sample, recorded by monitoring the emission at λ_em_ = 580 nm and under λ_exc_ = 360 nm excitation, respectively, optical absorption and 45 kV RL spectra of a representative Bi5 sample, illustrating that the X-ray-induced broad visible RL emission is spectrally separated from the strong near-band-edge absorption region, which can reduce reabsorption losses and favor visible-light extraction, X-ray irradiation stability of the representative double-sided ZnO:Bi nanorod scintillator prepared using a nominal Bi concentration of 10% (Bi10), RL spectra recorded at selected time intervals during 120 min of continuous X-ray irradiation at 45 kV and 200 μA, and integrated RL intensity as a function of irradiation time, normalized to the initial value (PDF)
