# Supplementary material for: Bi-Assisted Double-Sided ZnO Nanorod Architectures for Fast and Geometry-Enhanced X‑Ray Scintillation
Source: ACS Appl Opt Mater. 2026 Aug 25;4(9):2746–64. doi: 10.1021/acsaom.6c00409 (PMC13621809; doi:10.1021/acsaom.6c00409)
Supplement: Supplementary file 1 [file ot6c00409_si_001.pdf]

Supporting Information

Bi-Assisted Double-Sided ZnO Nanorod Architectures for Fast and Geometry-Enhanced X-ray Scintillation

Sinem V. Kurudirek[a], Anna Erickson[a], Paul J. Sellin[b], Benjamin J. Lawrie[c,d], Tanya A. Balandin[e], Luis Stand[f,g], Charles L. Melcher[f,g,h], Jason D. Azoulay[e], Nolan E. Hertel[a], Murat Kurudirek[a,b,i],*

[a] Nuclear and Radiological Engineering Program, G. W. Woodruff School of Mechanical Engineering, Georgia Institute of Technology, Atlanta, GA 30332, USA

[b] School of Mathematics and Physics, University of Surrey, Guildford GU2 7XH, UK

[c] Center for Nanophase Materials Sciences, Oak Ridge National Laboratory, Oak Ridge, TN 37831, USA

[d] Materials Science and Technology Division, Oak Ridge National Laboratory, Oak Ridge, TN 37831, USA

[e] School of Chemistry and Biochemistry, School of Materials Science and Engineering, Center for Organic Photonics and Electronics, Georgia Institute of Technology, Atlanta, GA 30332, USA

[f] Scintillation Materials Research Center, University of Tennessee, Knoxville, TN 37996, USA

[g] Department of Nuclear Engineering, University of Tennessee, Knoxville, TN 37996, USA

[h] Department of Materials Science and Engineering, University of Tennessee, Knoxville, TN 37996, USA

[i] Department of Electricity and Energy, Technical Sciences Vocational College, Ataturk University, 25240, Erzurum, Turkey

* *Corresponding author*: mkurudirek@atauni.edu.tr

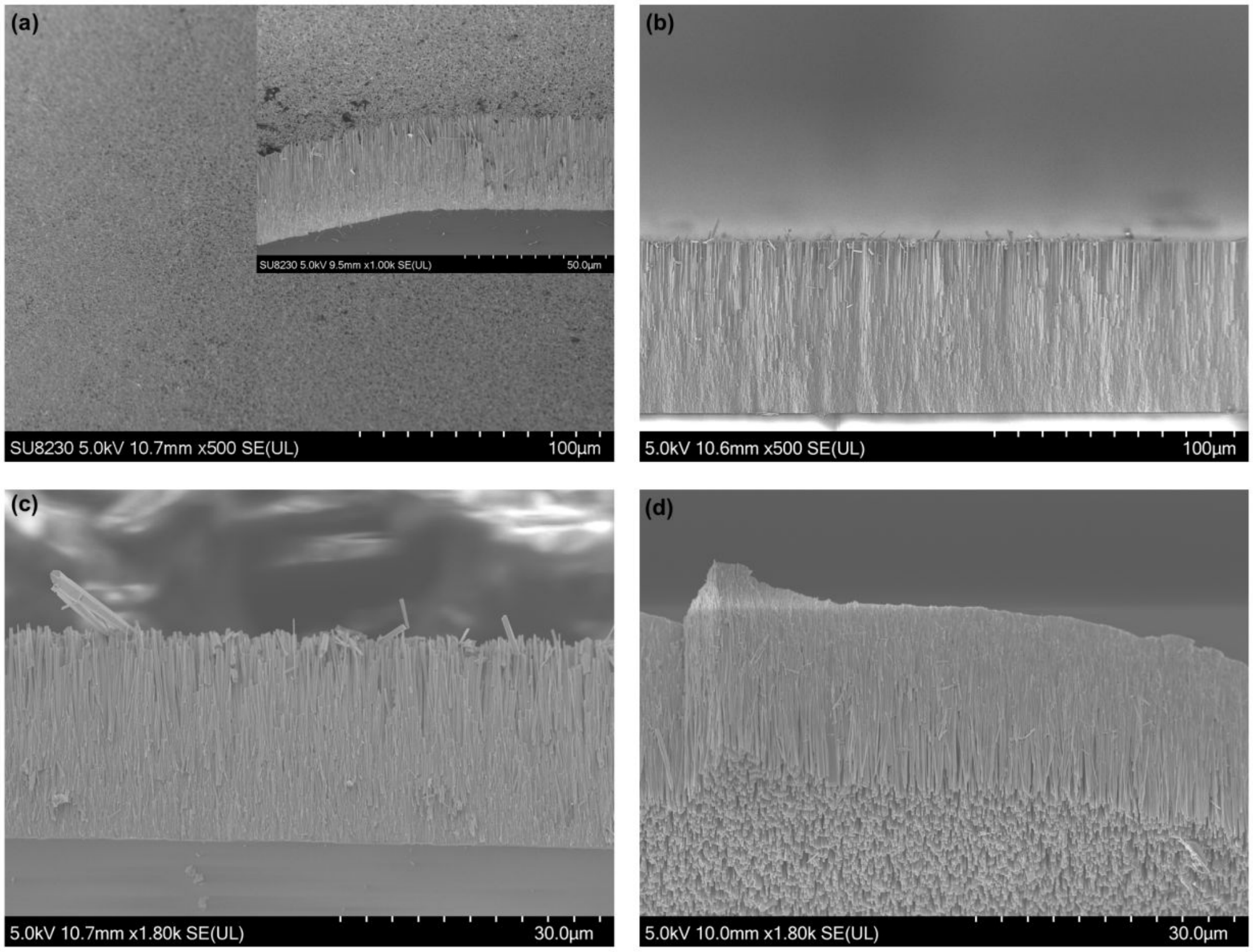

Figure S1. Additional SEM characterization of undoped, refresh-grown, and double-sided ZnO NR architectures. (a) Low-magnification top-view SEM image of undoped ZnO NR arrays with an inset showing the corresponding cross-sectional morphology. (b) Cross-sectional SEM image of the Bi-assisted refresh-grown ZnO NR layer, showing the formation of an extended high-aspect-ratio nanorod architecture. (c,d) Representative cross-sectional SEM images collected from opposite sides of the Bi-assisted double-sided ZnO NR architecture, supporting the formation of ZnO NR layers on both sides of the fused silica substrate.

Sample	Xc(002)	FWHM(002)	D(002)	Microstrain (002)	Xc(004)	FWHM(004)	D(004)	Microstrain (004)
UZO	34.48542	0.13113	63.5	0.00184	72.66032	0.25497	38.7	0.00151
Bi1	34.45109	0.07106	117.0	0.00100	72.6563	0.14344	68.7	0.00085
Bi5	34.46213	0.07945	104.7	0.00112	72.64928	0.15421	63.9	0.00092
Bi10	34.4567	0.05512	150.9	0.00078	72.65013	0.11002	89.6	0.00065
Ref5	34.42428	0.05967	139.4	0.00084	72.59335	0.07023	140.4	0.00042
Ref10	34.43969	0.0683	121.8	0.00096	72.60665	0.10636	92.7	0.00063
Ref20	34.75527	0.14692	56.7	0.00254	72.83639	0.14617	67.5	0.00086

Table S1. Peak fitting parameters and calculated structural quantities extracted from the ZnO (002) and (004) reflections, including peak center position, FWHM, crystallite size, and microstrain.

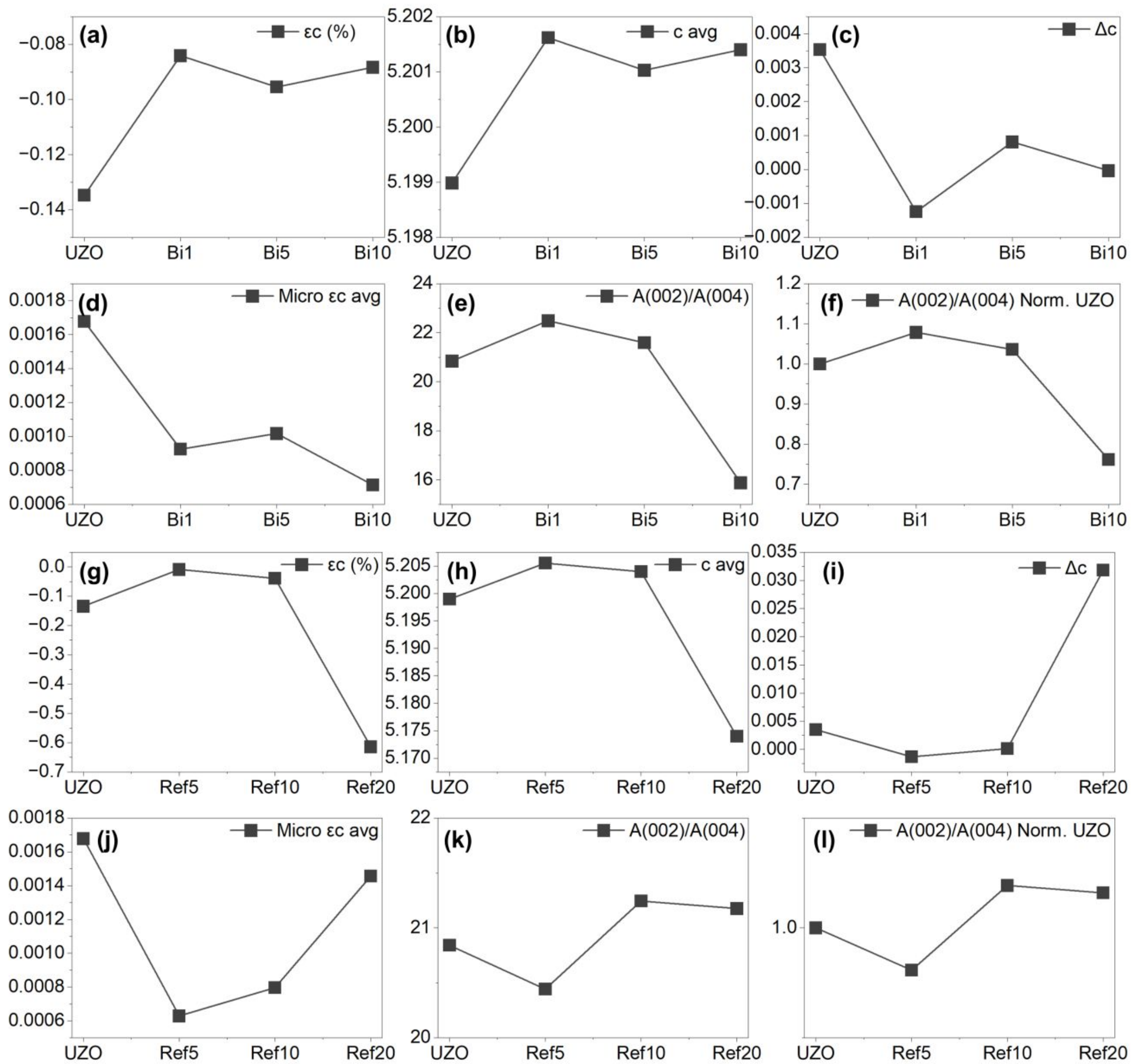

Figure S2. Additional XRD-derived structural parameters of Bi-assisted ZnO NR arrays. (a–f) Lattice strain-related parameter, average c-lattice parameter, Δc, average microstrain, A(002)/A(004) ratio, and normalized A(002)/A(004) ratio for undoped ZnO and Bi-assisted ZnO NR arrays. (g–l) Corresponding parameters for the refresh-grown Bi-assisted ZnO NR arrays. These additional parameters support the structural evolution discussed in the main text and indicate that low-to-moderate Bi incorporation modifies the c-axis-related structural properties while excessive nominal Bi loading leads to stronger structural perturbation.

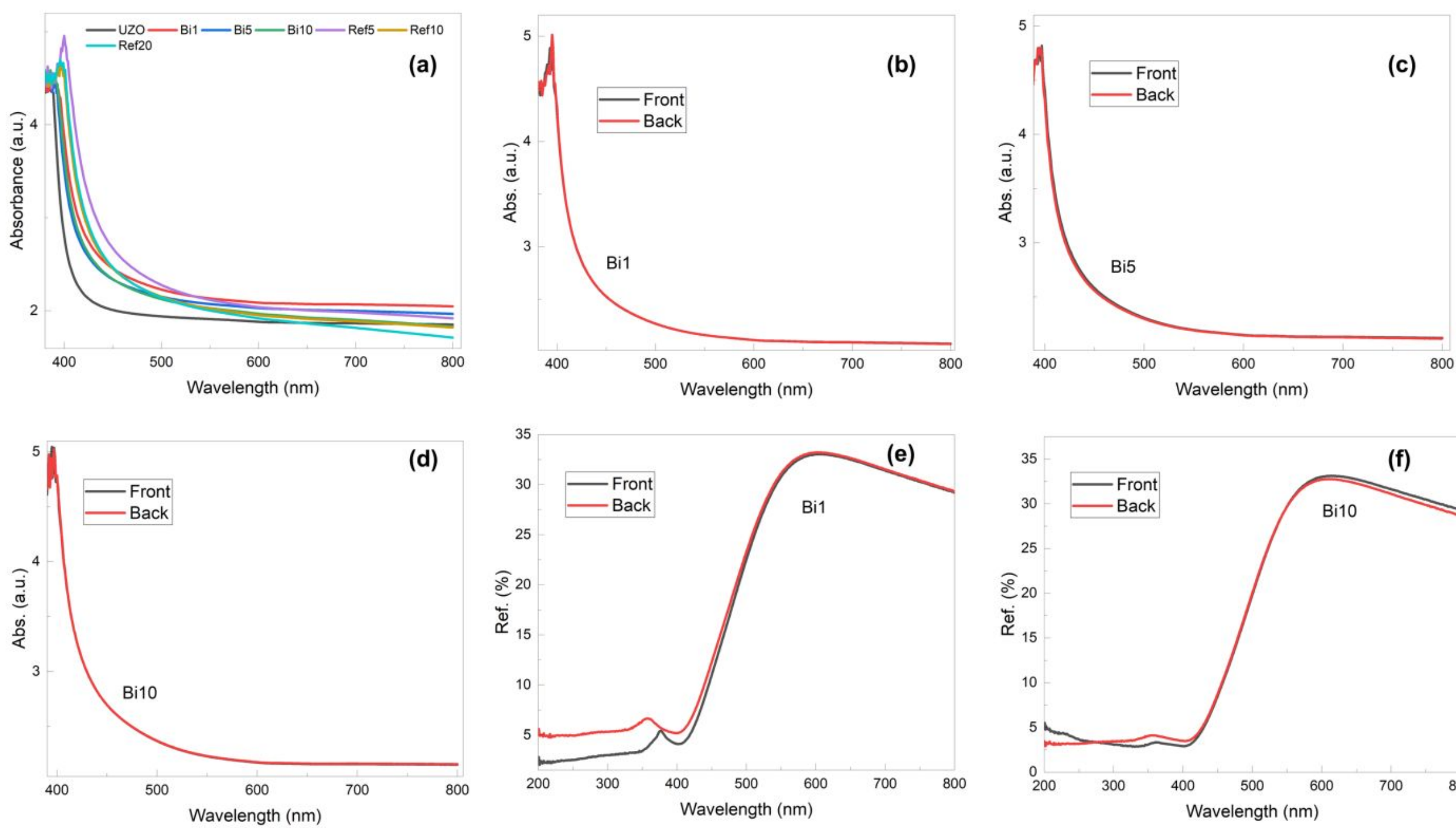

Figure S3. Supplementary UV–vis optical characterization of ZnO NR arrays. (a) Absorbance spectra of UZO, Bi-assisted ZnO, and refresh-grown ZnO NR samples. (b–d) UV–Vis absorbance spectra acquired in direct-transmission geometry from the two opposite orientations of the double-sided Bi1, Bi5, and Bi10 samples. (e–f) Front- and back-side diffuse reflectance spectra of

representative double-sided Bi1 and Bi10 samples, showing comparable optical response from both sides of the substrate.

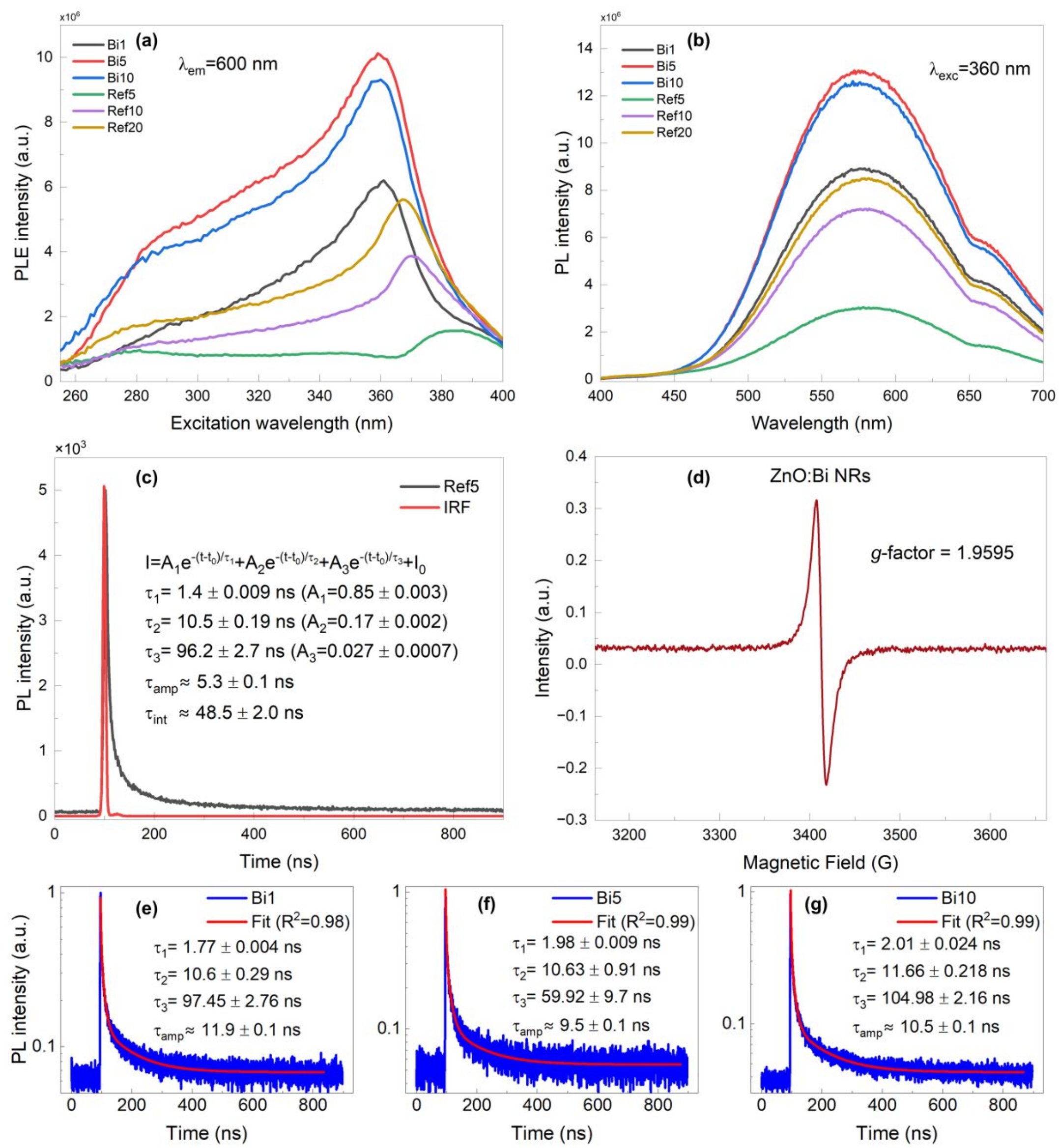

Figure S4. Additional optical and EPR characterization of ZnO:Bi NR arrays. (a) PLE spectra recorded by scanning the excitation wavelength while monitoring the emission at λ_{em} = 600 nm. (b) Emission spectra recorded under λ_{exc} = 360 nm excitation. (c) Time-resolved PL decay curve

of the representative Ref5 ZnO NR sample and the corresponding instrument response function (IRF). The Ref5 decay exhibits a pronounced tail extending well beyond the measured IRF, confirming that the overall response is not instrument-limited. The shortest fitted term is comparable to the temporal width of the IRF and is therefore not interpreted as an independently resolved intrinsic lifetime or assigned to a specific recombination process. (d) Room-temperature CW-EPR spectrum of the Bi-assisted ZnO NR sample. A clear resonance is observed at $g \approx 1.9595$, whereas no detectable signal is observed near $g \approx 2.0048$. At the applied microwave frequency, the $g \approx 2.0048$ resonance would be expected at approximately 3335 G. The magnetic field was calibrated and the sample was tuned prior to measurement. The EPR spectrum is interpreted qualitatively because the sample mass was not normalized to previous ZnO NR measurements. (e–g) Additional RT-TRPL decay curves and three-component exponential fits for representative ZnO:Bi NR samples, showing measurable nanosecond-scale decay dynamics with dominant fast components in the ~2–12 ns range and amplitude-weighted average lifetimes of approximately 9.5–11.9 ns.

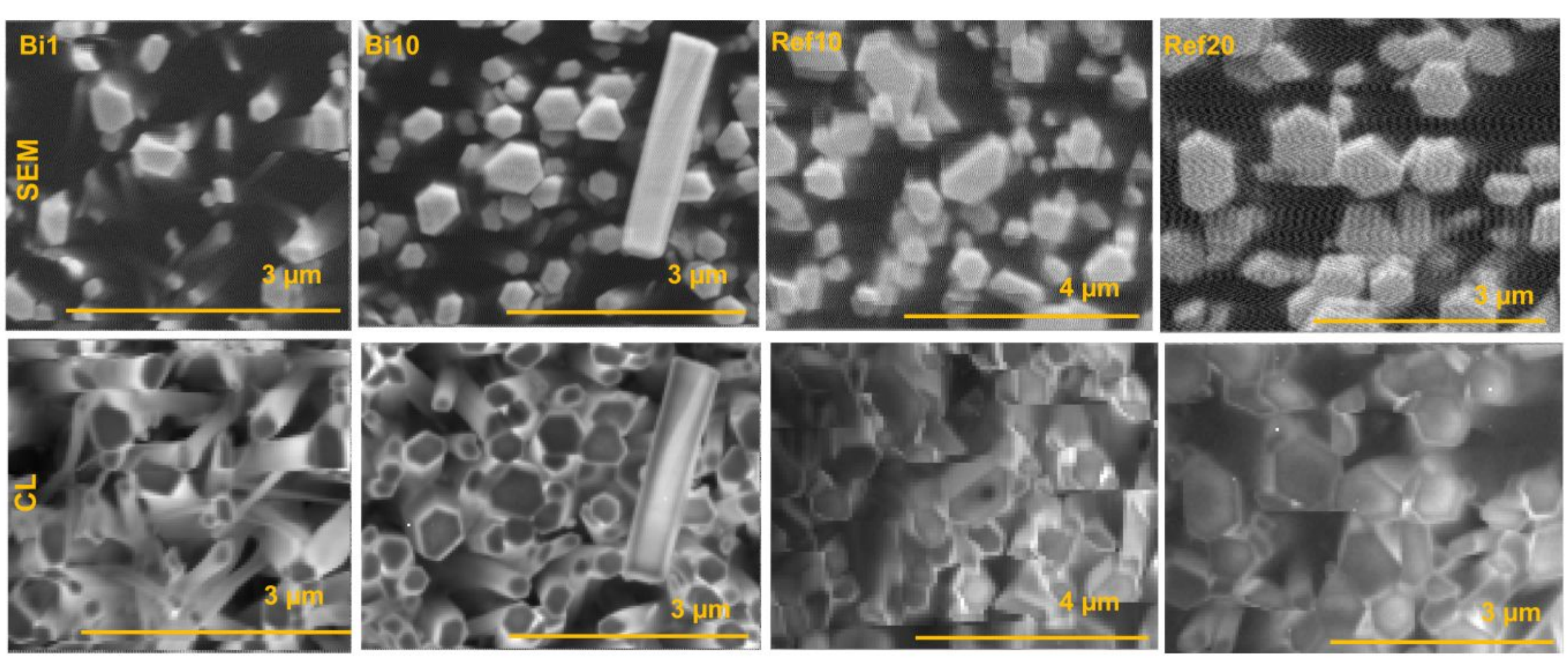

Figure S5. Representative SEM images and corresponding CL images of selected Bi-assisted and refresh-grown ZnO:Bi nanorod arrays. The CL images reveal spatially heterogeneous visible emission contrast, with enhanced emission frequently observed along nanorod edges and sidewall regions.

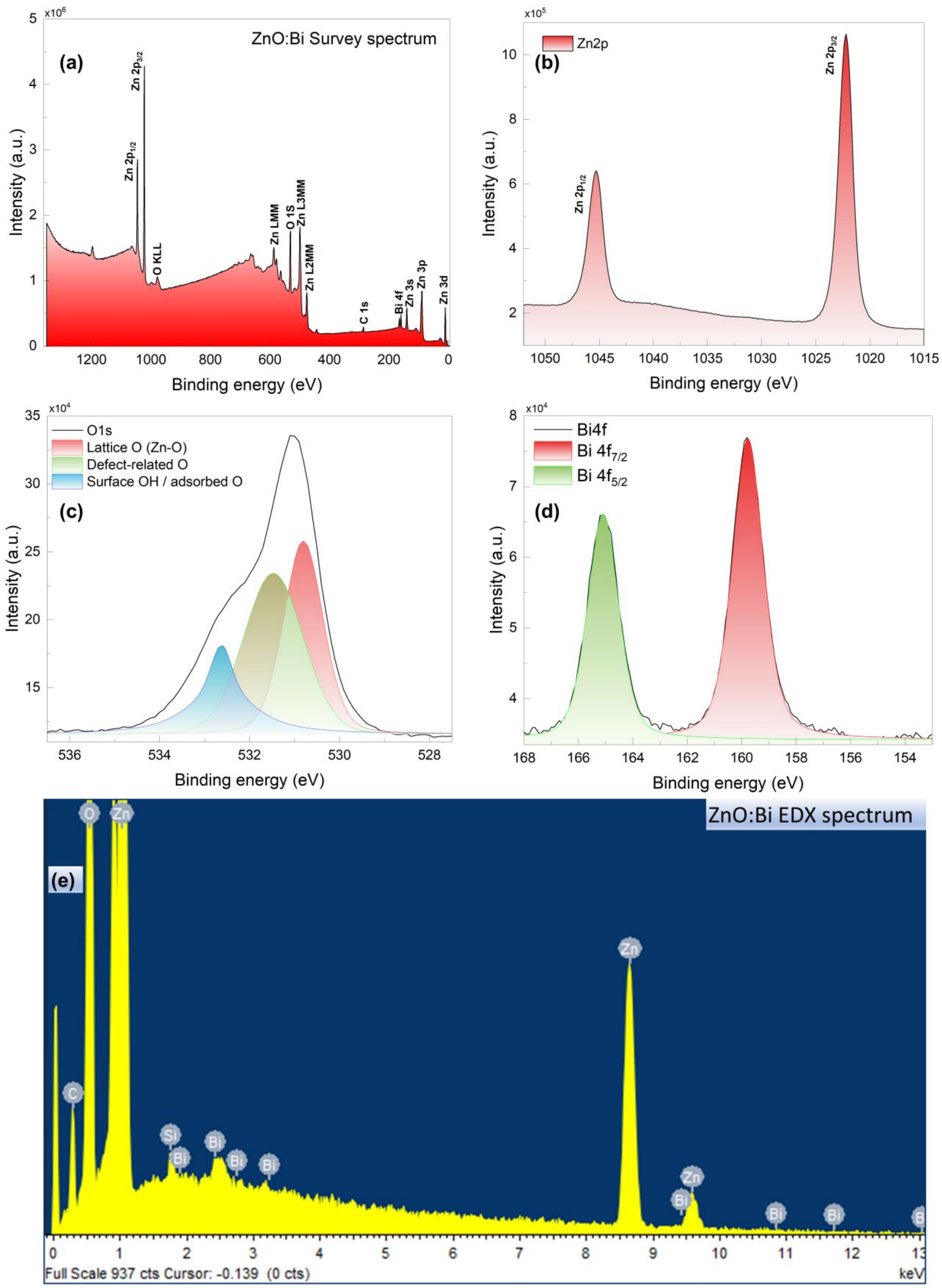
(a)
ZnO:Bi Survey spectrum
Zn 2p3/2
Zn 2p1/2
O KLL
Zn LMM
O 1S
Zn L3MM
Zn L2MM
C 1s
Bi 4f
Zn 3s
Zn 3p
Zn 3d
Intensity (a.u.)
Binding energy (eV)
(b)
Zn2p
Zn 2p1/2
Zn 2p3/2
(c)
O1s
Lattice O (Zn-O)
Defect-related O
Surface OH / adsorbed O
(d)
Bi4f
Bi 4f7/2
Bi 4f5/2
(e)
ZnO:Bi EDX spectrum
O
Zn
C
Si
Bi
Full Scale 937 cts Cursor: -0.139 (0 cts)
keV

Figure S6. XPS and EDX characterization of a representative ZnO:Bi nanorod sample. (a) XPS survey spectrum and high-resolution (b) Zn 2p, (c) O 1s, and (d) Bi 4f spectra. The O 1s spectrum was fitted with three components at ~530.8, ~531.5, and ~532.6 eV, corresponding to lattice oxygen in Zn–O, defect-related oxygen species, and surface hydroxyl/adsorbed oxygen species, respectively. The Bi 4f spectrum exhibits a spin–orbit doublet at ~159.8 and ~165.1 eV, assigned to Bi $4f_{7/2}$ and Bi $4f_{5/2}$, respectively; the splitting of ~5.3 eV and the fitted area ratio of ~1.38 are consistent with oxidized Bi species associated with the ZnO NR array. (e) EDX spectrum of the same representative ZnO:Bi sample, showing Bi-related peaks together with Zn and O signals.

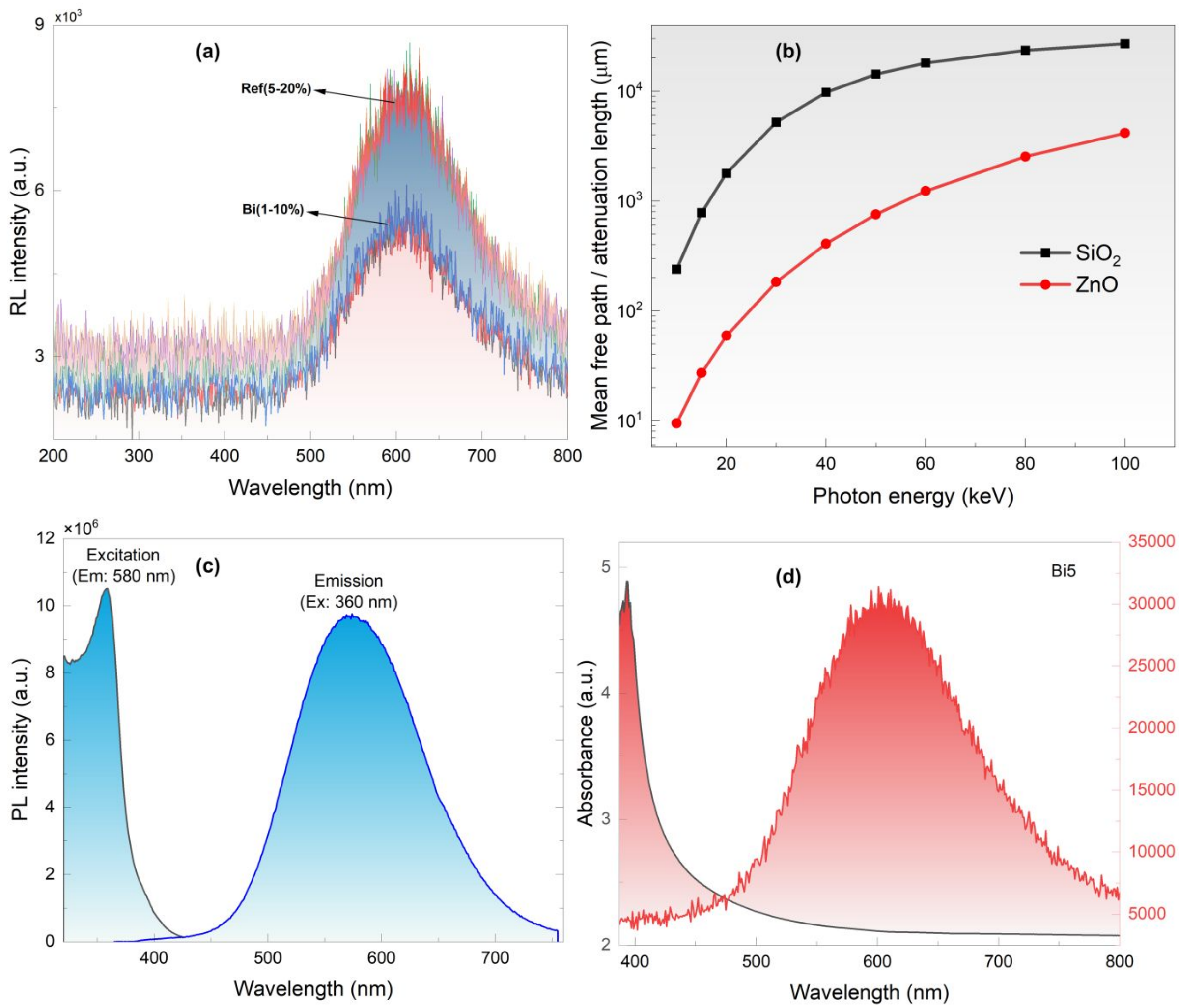

Figure S7. Supplementary optical emission, radioluminescence, and attenuation analysis of ZnO:Bi NR arrays. (a) RL spectra of single-sided ZnO:Bi and refresh-grown ZnO NR samples recorded under 35 kV X-ray tube voltage, showing broad yellow–orange emission centered at ~600 nm. (b) Calculated X-ray mean free path/attenuation length of SiO_2 and ZnO as a function of photon energy, supporting X-ray penetration through the fused-silica substrate in the 35–45 keV range. (c) Representative PLE and PL emission spectra of a ZnO:Bi NR sample, recorded by monitoring the emission at λ_{em} = 580 nm and under λ_{exc} = 360 nm excitation, respectively. The

spectra show efficient near-UV excitation and broad visible emission centered in the yellow–orange spectral region. (d) Optical absorption and 45 kV RL spectra of a representative Bi5 sample, illustrating that the X-ray-induced broad visible RL emission is spectrally separated from the strong near-band-edge absorption region, which can reduce reabsorption losses and favor visible-light extraction.

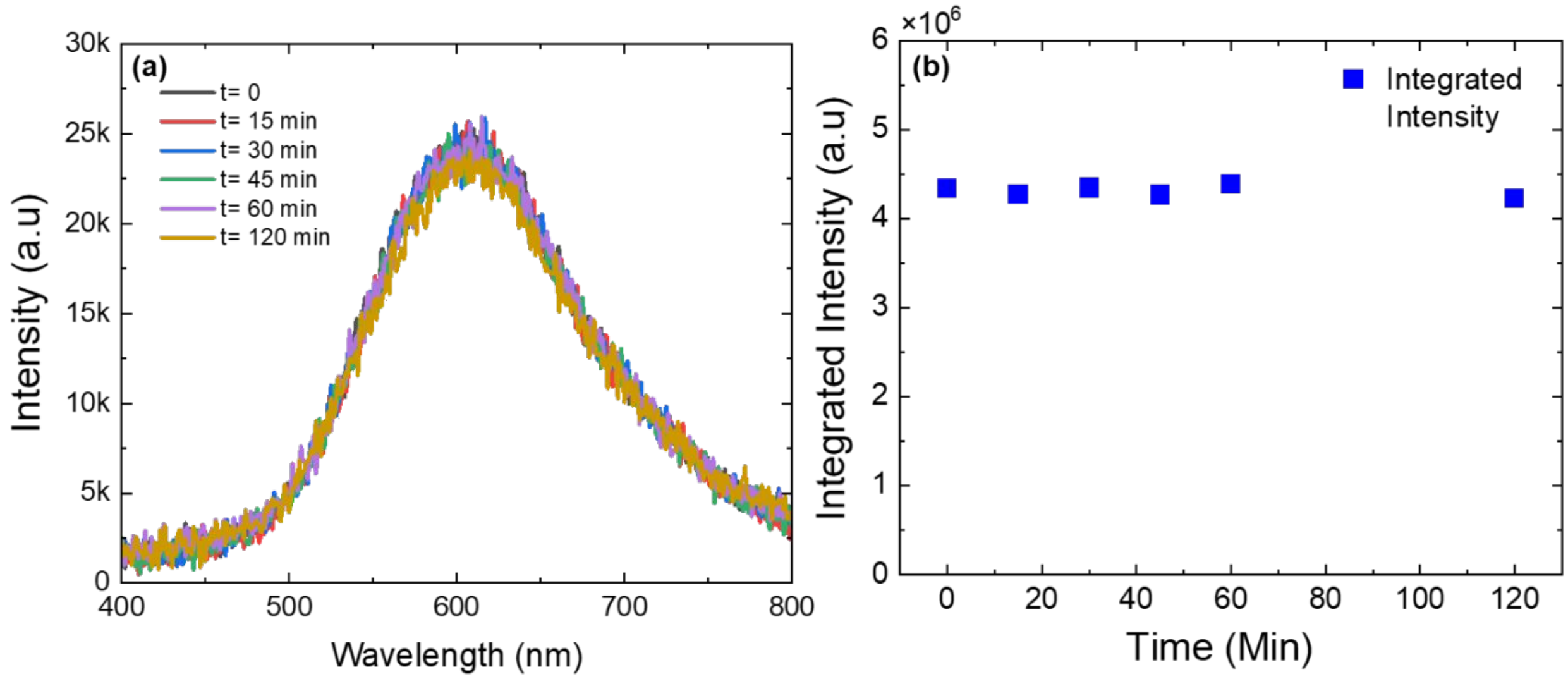

Figure S8. X-ray irradiation stability of the representative double-sided ZnO:Bi nanorod scintillator prepared using a nominal Bi concentration of 10% (Bi10). (a) RL spectra recorded at selected time intervals during 120 min of continuous X-ray irradiation at 45 kV and 200 μA. The spectrum labeled “Initial (0 min)” represents the initial measurement acquired at the beginning of irradiation. (b) Integrated RL intensity as a function of irradiation time.
